# Inter-isoform Hetero-dimerization of Human UDP-Glucuronosyltransferases (UGTs) 1A1, 1A9, and 2B7 and Impacts on Glucuronidation Activity

**DOI:** 10.1038/srep34450

**Published:** 2016-11-18

**Authors:** Ling-Min Yuan, Zhang-Zhao Gao, Hong-Ying Sun, Sai-Nan Qian, Yong-Sheng Xiao, Lian-Li Sun, Su Zeng

**Affiliations:** 1 Institute of Drug Metabolism and Pharmaceutical Analysis, Zhejiang Province Key Laboratory of Anti-Cancer Drug Research, College of Pharmaceutical Sciences, Zhejiang University, Hangzhou, 310058, China

## Abstract

Human UDP-glucuronosyltransferases (UGTs) play a pivotal role in phase II metabolism by catalyzing the glucuronidation of endobiotics and xenobiotics. The catalytic activities of UGTs are highly impacted by both genetic polymorphisms and oligomerization. The present study aimed to assess the inter-isoform hetero-dimerization of UGT1A1, 1A9, and 2B7, including the wild type (1A1*1, 1A9*1, and 2B7*1) and the naturally occurring (1A1*1b, 1A9*2/*3/*5, and 2B7*71S/*2/*5) variants. The related enzymes were double expressed in Bac-to-Bac systems. The fluorescence resonance energy transfer (FRET) technique and co-immunoprecipitation (Co-IP) revealed stable hetero-dimerization of UGT1A1, 1A9, and 2B7 allozymes. Variable FRET efficiencies and donor-acceptor distances suggested that genetic polymorphisms resulted in altered affinities to the target protein. In addition, the metabolic activities of UGTs were differentially altered upon hetero-dimerization via double expression systems. Moreover, protein interactions also changed the regioselectivity of UGT1A9 for querectin glucuronidation. These findings provide in-depth understanding of human UGT dimerization as well as clues for complicated UGT dependent metabolism in humans.

Human UDP-glucuronosyltransferases (UGTs) are key phase II metabolism related enzymes, which conjugate various endobiotics and xenobiotics with the glucuronic acid group of uridine diphosphoglucuronic acid (UDPGA). Given their high polarity, glucuronides are excreted in the urine or bile^1^,^2^. Thereby, glucuronidation catalyzed by human UGTs represents one of the most important detoxifying processes. To date, nineteen functionally active human UGTs have been identified, and are divided into three subfamilies (UGT1A, 2A, and 2B) based on evolutionary divergence^3^. Of the human UGT members, UGT1A1, 1A9 and 2B7 possess significant catalyzing capacities, and play key roles in the metabolism of therapeutic and environmental compounds, as well as endobiotics such as steroids, fatty acids, and bilirubin^2^. With respect to substrate selectivity, UGT enzymes display distinct, but overlapping functions. Representative substrates of UGT1A1 include bilirubin, ethynylestradiol, raloxifene, and irinotecan^4^,^5^,^6^. Meanwhile, estrone, mycophenolic acid, raloxifene and retinoic acid are typical substrates for UGT1A9^5^,^7^,^8^,^9^. UGT2B7 is known as the most important human UGT enzyme responsible for phase II metabolism of most clinically used drugs, including morphine, zidovudine, and carbamazepine^10^,^11^,^12^,^13^.

As previously reported, human UGTs are coded by polymorphic genes, and the polymorphism effects on metabolism have been comprehensively investigated^14^,^15^,^16^. UGT1A1, 1A9 and 2B7 are considered highly polymorphic enzymes. UGT1A1 is the only identified UGT enzyme with bilirubin biotransformation activity. A slightly active variant of UGT1A1 causes bilirubin accumulation, resulting in fatal hyperbilirubinemia^4^. UGT1A9 acts as the predominant enzyme to terminate the biological activity of mycophenolic acid, which is the active metabolite of mycophenolate mofetil, a commonly used immunosuppressant and antiproliferative agent for transplantation patients^17^. The metabolic rates of UGT1A9 allozymes toward mycophenolic acid display a 3-fold variability^18^. UGT2B7 polymorphism significantly alters clearance and outcomes of therapeutic drugs^19^,^20^. Moreover, individuals harboring the UGT2B7*2 allele have an increased risk of certain cancers, e.g. bladder cancer^21^.

In addition to polymorphisms, oligomerization is another feature of human UGTs. Direct evidences of human UGTs forming various homo- and hetero-oligomers were obtained by co-immunoprecipitation (Co-IP) and fluorescence resonance energy transfer (FRET) techniques. UGT2B1 is known to form complexes with UGT1A proteins^22^. UGT1A members are capable of forming dimers within the enzyme family^23^. Fujiwara *et al*. confirmed the existence of protein-protein interactions between wild type UGT2B7 and several UGT1A members, using Co-IP^24^. Homo- and hetero-dimerization of UGT1A1 and UGT1A9 allozymes^25^, as well as UGT2B7 allozymes^12^, have recently been reported by our research team. Furthermore, increasing evidence suggests that oligomerization remarkably affects the catalytic activity of individual enzymes involved in the oligomerization system. Dimerization of two different inactive mutants of UGT2B1 results in enzymatic activity recovery^26^. Double expressed wild type UGT2B7 with its allelic variants show significant differences in kinetic behaviors for zidovudine glucuronidation, compared with the single expressed form^12^. Therefore, protein-protein interactions of UGTs play key roles in glucuronidation activity regulation.

The current study aimed to assess inter-isoform hetero-dimerization among UGT1A1, 1A9 and 2B7. The studied enzymes included wild types (*1), one splice mutant of UGT1A1 (1A1*1b), three allelic variants of UGT1A9 (1A9*2, *3, and *5), and three UGT2B7 mutants (2B7*71S, *2, and *5). The Bac-to-Bac system has been used for single or dual expression of target UGT enzymes. We applied quantitative FRET and Co-IP for assessing hetero-dimerization among UGT1A1, 1A9, and 2B7. To assess the relationships between dimerization and glucuronidation activities, quercetin and zidovudine were selected as probe substrates to characterize the catalytic patterns of heterodimers.

## Results

### FRET analysis

**Figure 1:**
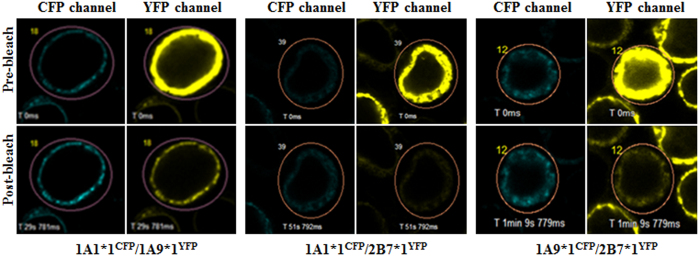
FRET images of Sf9 cells co-infected with recombinant UGT1A1*1^CFP^/1A9*1^YFP^, UGT1A1*1^CFP^/2B7*1^YFP^, and UGT1A9*1^CFP^/2B7*1^YFP^, presented as typical FRET detection using the acceptor photobleaching method. Increased CFP fluorescence in the circled area by 515 nm laser line bleaching indicated interactions between the proteins.

Hetero-dimerization of UGT1A1*N/1A9*N, UGT1A1*N/2B7*N, and UGT1A9*N/2B7*N was analyzed by quantitative FRET, a reliable method for assessing intra- and inter-molecular interactions^27^,^28^,^29^. CFP-tagged UGT1A1*N and 1A9*N, and YFP-tagged UGT1A9*N and 2B7*N enzymes were individually detected with both CFP and YFP channels, and fluorescence tagged UGTs were stably expressed in the endoplasmic reticulum (ER) membrane (Fig. S1). Consequently, recombinant UGT enzymes were double expressed in Sf9 cells as demonstrated by fluorescence microscopy. Sf9 cells co-infected with *CFP*/*YFP* baculovirus and those infected with *CFP-linker-YFP* baculovirus served as negative and positive controls, respectively. We employed the acceptor photobleaching method to determine the FRET phenomenon. After photobleaching the acceptor YFP, no increase of CFP signals in the negative control (Fig. S2A) was observed; meanwhile, a significant increase occurred in the positive control (Fig. S2B). Except UGT1A1*1b^CFP^/2B7*5^YFP^, all the tested combinations of UGT1A1*N, 1A9*N and 2B7*N exhibited the FRET phenomenon in the co-expression system (Fig. 1); these findings suggested that the two enzymes resided in a close distance and formed protein-protein interactions.

The affinity among UGT isoforms can be quantitatively evaluated by FRET efficiency (E%) and donor-acceptor distance (*r*). E% of the dimers varied from −0.74% to 24.3% (Table 1), while *r* values ranged from 6.38 nm to more than 10 nm. The higher FRET efficiency and shorter donor-acceptor distance both indicated higher interaction ability with the target protein. Comparing the obtained E% and *r* values, UGT1A1*1^CFP^ and 1A9*3^YFP^ showed the highest affinity with each other, while UGT1A1*1b^CFP^ and 2B7*5^YFP^ displayed the lowest. Compared with wild type UGT1A1 involved dimeric complexes, FRET efficiencies of dimers formed with the splice mutant UGT1A1*1b were dramatically decreased, implying weaker interactions. Based on highly altered FRET efficiencies, the mutation sites in UGT1A9 (*2, *3, and *5) and UGT2B7 (*71S, *2, and *5) variants all influenced the protein-protein interactions.

### Co-immunoprecipitation

**Figure 2:**
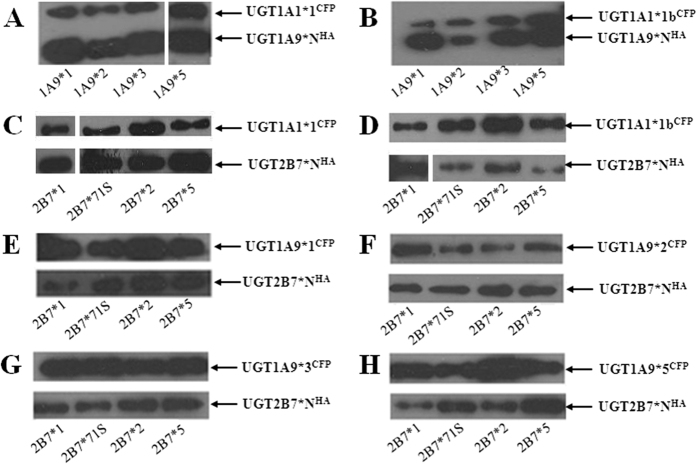
Co-IP analysis of cell lysates from UGT1A1, 1A9, and 2B7 double expression systems. Sf9 cells were co-infected with UGT1A1*N^CFP^/1A9*N^HA^, UGT1A1*N^CFP^/2B7*N^HA^, or UGT1A9*N^CFP^/2B7*N^HA^ baculovirus. Cell lysates from double expression systems were immunoprecipitated with anti-HA beads followed by Western blot analysis using anti-UGT1A and anti-UGT2B7 antibodies. Images A to H represent Western blot results of cell lysates from UGTA1*N^CFP^/1A9*N^HA^, UGTA1*N^CFP^/2B7*N^HA^, UGTA9*N^CFP^/2B7*N^HA^ double expression systems. The full-length blots are presented in Fig. S4.

In addition to FRET, hetero-dimerization of UGTs was confirmed by conventional co-immunoprecipitation (Co-IP). Fusion proteins, including UGT1A1*N^CFP^, UGT1A9*N^CFP^, UGT1A9*N^HA^ and UGT2B7*N^HA^, were used for Co-IP assays (Fig. S3). Anti-HA beads were used to immunoprecipitate HA-tagged UGT1A9 and UGT2B7 allozymes. Co-IP experiments were performed with mixtures of two different UGT single expression systems, and alternatively, with UGT double expression systems. For the former operation, cell lysates from UGT1A1*N^CFP^ (81 kDa) or UGT1A9*N^CFP^ (81 kDa) single expression system were mixed with those of UGT1A9*N^HA^ (56 kDa) or UGT2B7*N^HA^ (56 kDa). Subsequently, the mixtures were immunoprecipitated with anti-HA beads and detected by Western blot. As shown in Fig. S5, the bands corresponding to UGT1A1*N^CFP^ or UGT1A9*N^CFP^ did not appear after Co-IP. Only HA-tagged UGT1A9 and UGT2B7 allozymes were detectable. Meanwhile, in the second Co-IP procedure, cell lysates from CFP-tagged and HA-tagged UGTs double expression systems were processed for anti-HA bead immunoprecipitation and Western blot analysis. Both bands of CFP-tagged (81 kDa) and HA-tagged (56 kDa) UGTs were found in Western blot experiments (Fig. 2). These findings indicated that HA-tagged UGT enzymes were immunoprecipitated with CFP-tagged proteins, and hetero-dimers existed. Notably, without a significant FRET phenomena (FRET efficiency was −0.74%, the donor-acceptor distance >10 nm.), hetero-dimerization of UGT1A1*1b and 2B7*5 remained undefined. Nevertheless, based on Co-IP analysis, the two proteins still formed a complex during co-expression. The obtained results implied that co-expression, or intracellular environment was an essential condition for the hetero-dimerization of UGT1A1, 1A9 and 2B7.

### Catalytic analysis of UGT hetero-dimers

#### Relative expression levels of UGTs in double expression systems

To normalize the catalytic activity assay, relative expression levels of UGTs in double expression systems were determined by immunoblot. UGT1A1 and 1A9 allozymes were detected by anti-UGT1A antibodies, while UGT2B7 allozymes were selectively probed by anti-UGT2B7 antibodies (Figs S6–S8). The expression levels of singly expressed UGT1A1*1^HA^ and UGT2B7*1^HA^ were defined as 100%. Relative expression levels of UGT1A1*N in double expression systems varied from 26.7% to 230.8% (Figs S9 and S10A). Compared with 38.5~211.6% relative expression levels in co-expression systems with UGT1A1*N (Fig. S9), UGT1A9*N allozymes expression was reduced in co-expression systems with UGT2B7*N, ranging from 12% to 81% (Fig. S11A). Expression levels of UGT2B7 allozymes co-expressed with UGT1A9 allozymes (45~231%) (Fig. S11B) were remarkably higher than when co-expressed with UGT1A1 allozymes (11~63%) (Fig. S10B). Relative expression levels were used to correct enzyme activity.

#### Quercetin glucuronidation

To assess the effects of protein-protein interactions on enzymatic activity of UGT1A1 and UGT1A9, quercetin glucuronidation activity of double expression systems was evaluated^30^. Glucuronides were detected and quantified by a modified HPLC method^25^. UGT1A1 mainly converted quercetin into three monoglucuronides, including M1 (7-glucuronide), M3 (4′-glucuronide), and M4 (3′-glucuronide). The major products of UGT1A9 catalyzed quercetin glucuronidation were M1, M2 (3-glucuronide), and M4. UGT1A1*1b is the non-functional splice mutant. Additionally, we confirmed that UGT2B7 was not capable of converting quercetin.

**Figure 3:**
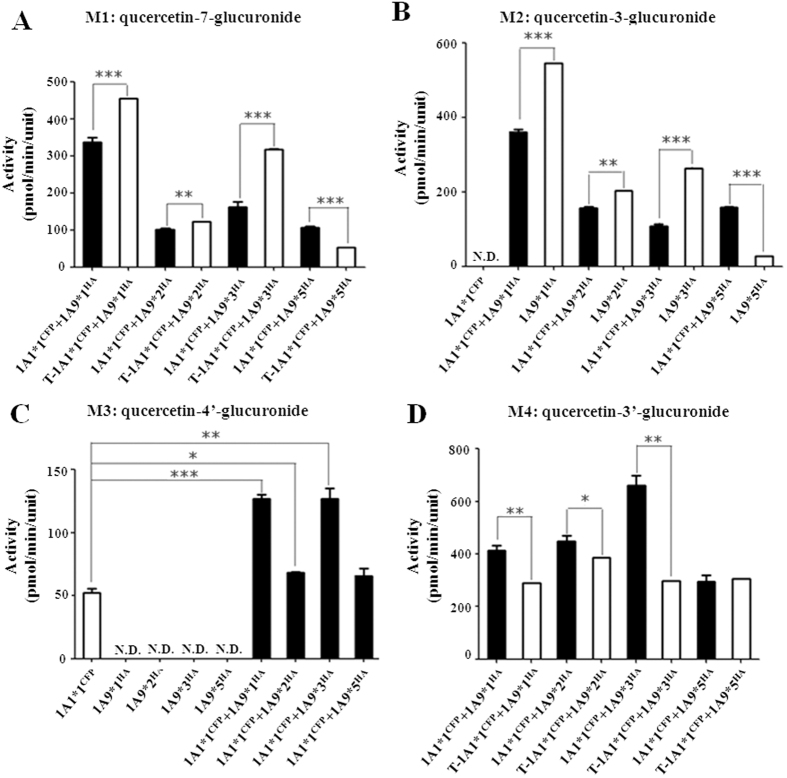
Quercetin glucuronidation assays of UGT1A1*1^CFP^/1A9*N^HA^ double expression systems. Concentration of quercetin was 100 μM ◻ and ◼ indicate single and double expressions, respectively. N.D., not detectable. Data are mean ± SD from triplicate experiments. Asterisks indicate statistically significant differences (***P < 0.0001; **P < 0.005; *P < 0.05). T-UGT1A1*1^CFP^ + UGT1A9*N^HA^ indicates the algebraic sum of the theoretical activity of single expressed UGT1A1*1 and UGT1A9*N.

**Figure 4:**
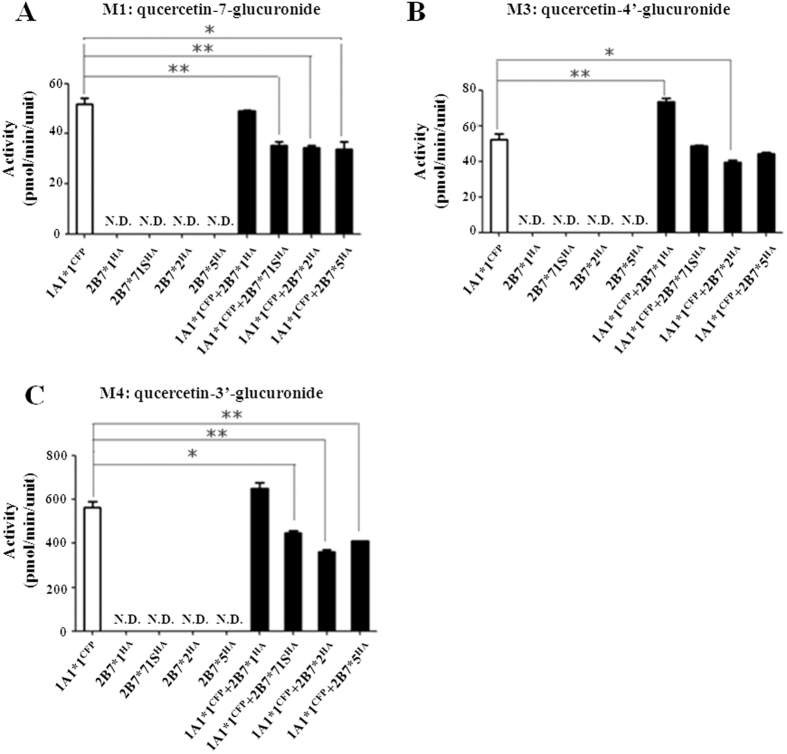
Quercetin glucuronidation assays of UGT1A1*1^CFP^/2B7*N^HA^ double expression systems. Concentration of quercetin was 100 μM ◻ and ◼ indicate single and double expressions, respectively. N.D., not detectable. Data are mean ± SD from triplicate experiments. Asterisks indicate statistically significant differences (***P < 0.0001; **P < 0.005; *P < 0.05).

As M1 and M4 are common quercetin metabolic products of UGT1A1 and 1A9, the formation of these two glucuronides resulted from both enzymes in UGT1A1*1/1A9*N double expression system. With the exception of the UGT1A1*1/1A9*5 dimer that showed significantly higher M1 formation activity compared with the algebraic sum of theoretical activity of two single expressed enzymes, hetero-dimerization of UGT1A1*1 with UGT1A9*1, *2 or *3 decreased M1 production (Fig. 3A). In contrast, activity toward M4 was mostly enhanced in UGT1A1*1/1A9*N dimers (Fig. 3D). Deduced from M3 formation, which was specifically catalyzed by UGT1A1, co-expression with UGT1A9*2 or *3 elevated the activity of UGT1A1*1 by nearly 2-fold, while co-expression with UGT1A9*1 or *5 showed a similar activity with the single expressed UGT1A1*1 (Fig. 3C). Through M2 formation, hetero-dimerization with UGT1A1*1 decreased UGT1A9*1, *2, and *3 activities, but dramatically increased that of UGT1A9*5 (Fig. 3B). Moreover, compared with UGT1A1*1 single expressed enzyme, interactions with UGT2B7*1 resulted in enhanced M3 formation, with minor influence on M1 and M4 formation. Meanwhile interactions with UGT2B7 variants (*71S, *2 and *5) inhibited the productions of all three metabolites by UGT1A1 (Fig. 4).

**Figure 5:**
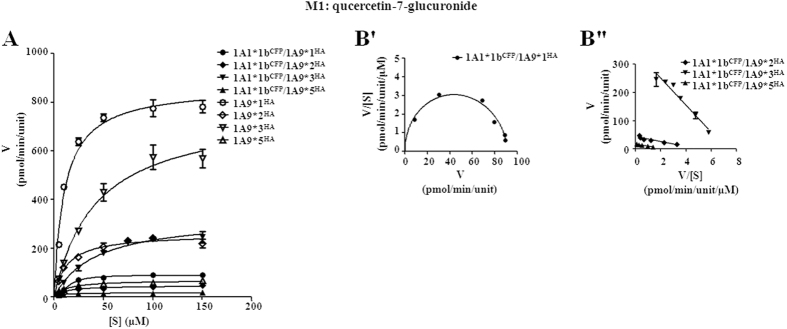
Enzyme kinetic plots (**A**), V/[S]-V plots (**B′**) and Eadie-Hofstee plots (**B″**) of M1 (quercetin-7-glucuronide) formation by UGT1A1*1b^CFP^/1A9*N^HA^ co-expression systems. Glucuronidation rates are mean ± SD of three independent determinations, and normalized according to relative expression levels. The respective kinetic constants are presented in Table 2.

**Figure 6:**
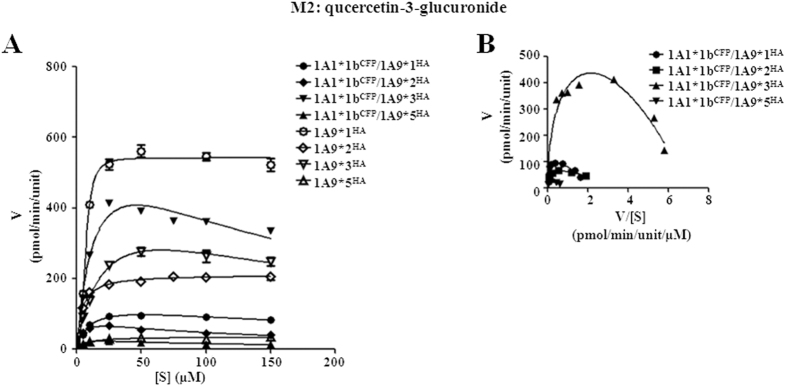
Enzyme kinetic plots (**A**), and Eadie-Hofstee plots (**B**) of M2 (quercetin-3-glucuronide) formation by UGT1A1*1b^CFP^/1A9*N^HA^ co-expression systems. Glucuronidation rates are mean ± SD of three independent determinations, and normalized according to relative expression levels. The respective kinetic constants are presented in Table 3.

The impacts of hetero-dimerization with UGT1A1*1b on UGT1A9*N activity were determined by kinetic assessments. As shown in Figs 5 and 6, only M1 formation kinetics of UGT1A9*2, *3, or *5 dimers with 1A1*1b fitted well with Michaelis-Menten kinetics. Concerning *CL*_int_ values, interactions with UGT1A1*1b inhibited M1 and M4 formation activity of the entire UGT1A9*N group to less than 45% compared with the corresponding single enzyme, and severely impaired the activity of wild type UGT1A9 to ~7% (Table 2). For M2 formation, binding with UGT1A1*1b greatly enhanced the activity of UGT1A9*1 and *2 to 212% and 154%, respectively. However, an opposite effect was observed for UGT1A9*3 and *5, in both of which *CL*_int_ was decreased to around 35% of single enzyme value (Table 3). During M1 formation, only UGT1A1*1b/1A9*1 dimer exhibited substrate inhibition, whereas in M2 production, all the four UGT1A1*1b/1A9*N dimers displayed this feature.

**Figure 7:**
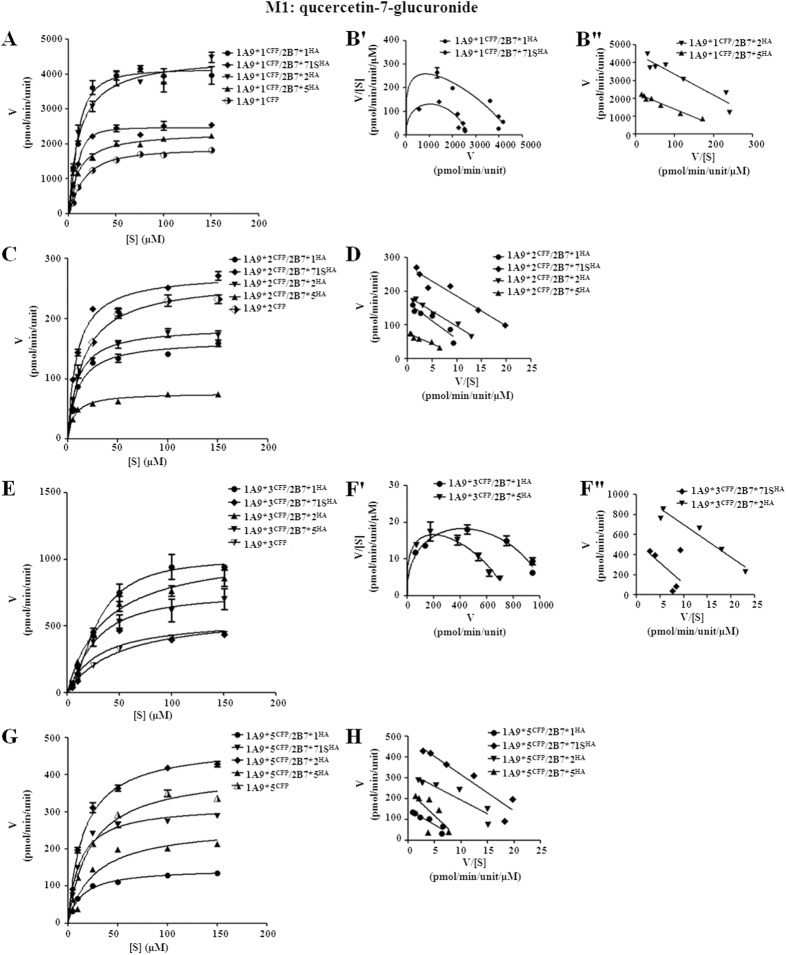
Enzyme kinetic plots (**A,C,E,G**), V/[S]-V plots (**B,F′**) and Eadie-Hofstee plots (**B″,D,F″,H**) of M1 (quercetin-7-glucuronide) formation by UGT1A9*N^CFP^/2B7*N^HA^ co-expression systems. Glucuronidation rates are mean ± SD of three independent determinations, and normalized according to relative expression levels. The respective kinetic constants are presented in Table 4.

**Figure 8:**
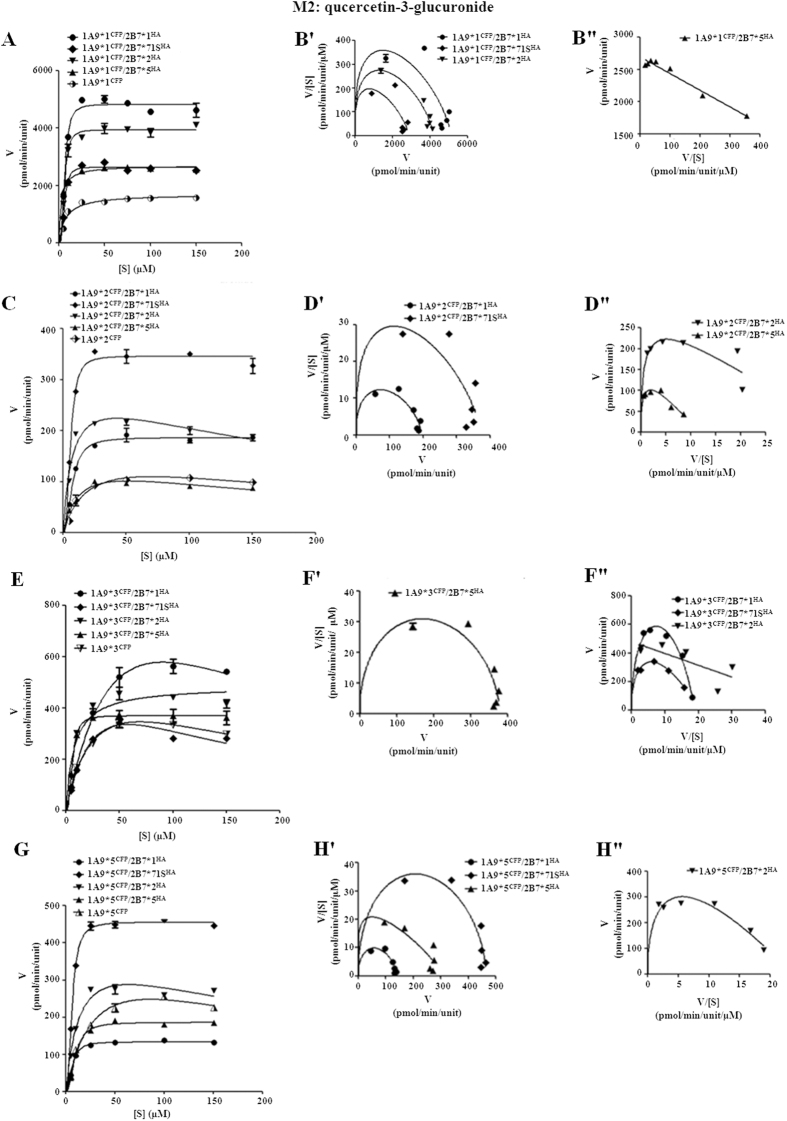
Enzyme kinetic plots (**A,C,E,G**), V/[S]-V plots (**B′,D′,F′,H′**) and Eadie-Hofstee plots (**B″,D″,F″,H″**) of M2 (quercetin-3-glucuronide) formation by UGT1A9*N^CFP^/2B7*N^HA^ co-expression systems. Glucuronidation rates are mean ± SD of three independent determinations, and normalized according to relative expression levels. The respective kinetic constants are presented in Table 5.

Kinetic behaviors of M1 and M2 formation by UGT1A9*N/2B7*N dual expression systems were also comprehensively investigated. In M1 formation, except UGT1A9*1/2B7*1, UGT1A9*1/2B7*71S, UGT1A9*3/2B7*1, and UGT1A9*3/2B7*5 dimers, the kinetic parameters of the remaining UGT1A9*N/2B7*N dimers fitted well to Michaelis-Menten kinetics (Fig. 7). In M2 formation, among all the tested hetero-dimers, only UGT1A9*1/2B7*1 followed the Michaelis-Menten kinetics (Fig. 8). Co-expression with UGT2B7 allozymes, including UGT1A9*1 and *3, resulted in increased M1 formation activity with relative *CL*_int_ values ranging from 132% to 579%. However, the dimerization effects of the UGT2B7 allozymes on UGT1A9*2 and *5 were greatly variable (Table 4). Thirteen out of sixteen tested UGT1A9*N/2B7*N double expression models exhibited markedly increased M2 formation activity compared with the corresponding single expressed UGT1A9 allozymes (Table 5); specifically, UGT1A9*1/2B7*5 complex had an *CL*_int_ value increased by nearly 9-fold. The UGT1A9*5/2B7*1 dimer was the only case with a reduced M2 formation activity (~75%). Meanwhile, UGT1A9*3 co-expressed with UGT2B7*1 or 2B7*71S retained a *CL*_int_ similar to that of its single expression form. Additionally, the effects of hetero-dimerization on M4 formation of UGT1A9*N were determined by relative activity assay. Interactions with UGT1A1*1b severely impaired the UGT1A9*N activity toward M4 (Fig. S12), whereas hetero-dimerization with UGT2B7*N up-regulated M4 formation (Fig. S13). Notably, in M2 formation, substrate inhibition was observed in four UGT1A9*N/2B7*N double expression systems, including UGT1A9*2/2B7*2, 1A9*2/2B7*5, 1A9*3/2B7*1, and 1A9*3/2B7*71S (Table 5).

#### Zidovudine O-glucuronidation

**Figure 9:**
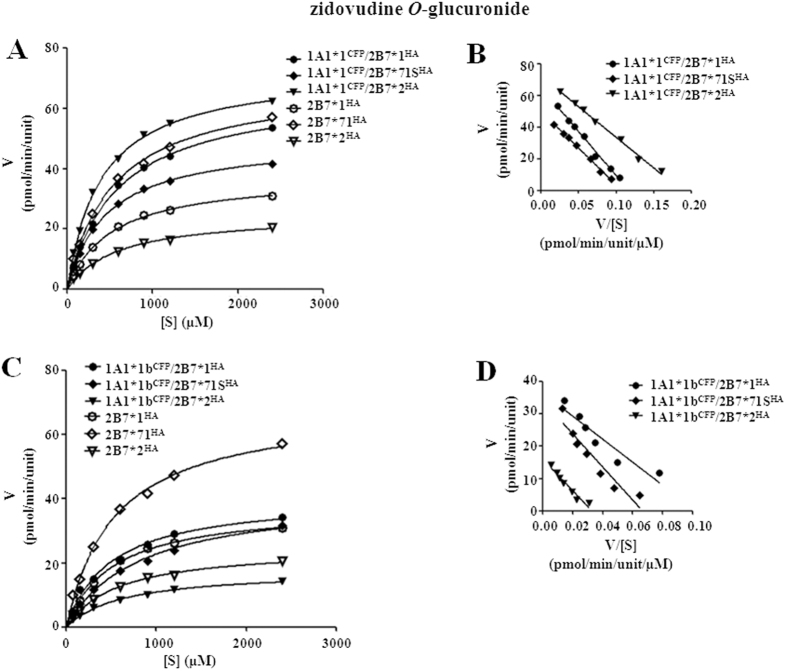
Enzyme kinetic plots (**A,C**) and Eadie-Hofstee plots (**B,D**) of zidovudine *O*-glucuronidation by UGT1A1*N^CFP^/2B7*N^HA^ co-expression systems. Glucuronidation rates are mean ± SD of three independent determinations, and normalized according to relative expression levels. The respective kinetic constants are presented in Table 6.

**Figure 10:**
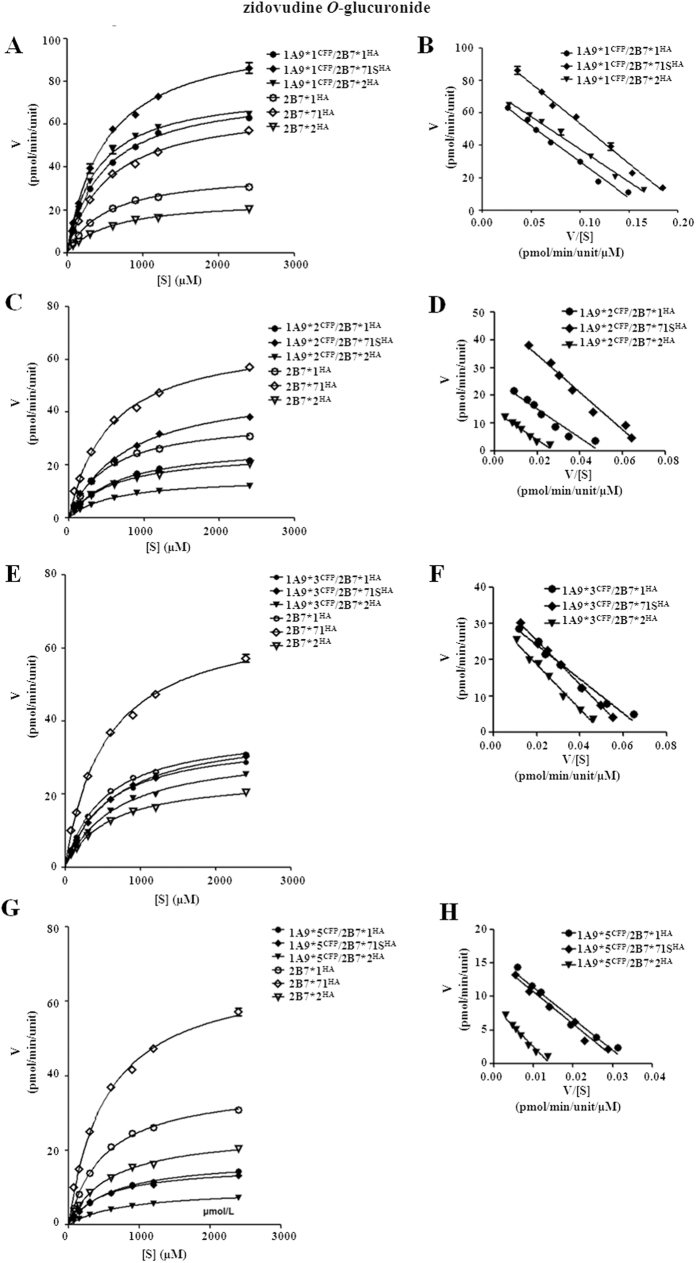
Enzyme kinetic plots (**A,C,E,G**) and Eadie-Hofstee plots (**B,D,F,H**) of zidovudine *O*-glucuronidation by UGT1A9*N^CFP^/2B7*N^HA^ co-expression systems. Glucuronidation rates are mean ± SD of three independent determinations, and normalized according to relative expression levels. The respective kinetic constants are presented in Table 7.

As an UGT2B7 specific catalyzing reaction, zidovudine glucuronidation was detected for assessing the activity of UGT2B7 involved double expression systems. UGT1A1 and UGT1A9 allozymes, as well as their hetero-dimers with the inactive UGT2B7*5 mutant, showed no detectable zidovudine glucuronidation activity. Overall, all UGT1A1*N/2B7*N and UGT1A9*N/2B7*N hetero-dimers showed Michaelis-Menten kinetics (Figs 9 and 10). In terms of *CL*_int_ values, UGT2B7*N dimer with UGT1A1*1 had markedly higher activity than the corresponding dimer with the UGT1A1*1b mutant (Table 6). For instance, dimerization with UGT1A1*1 resulted in >4-fold higher activity compared with single expressed UGT2B7*2; however, dimerization with UGT1A1*1b caused UGT2B7*2 activity to decrease to ~60%. In the UGT1A9*N/2B7*N group, protein interactions with wild type UGT1A9 resulted in greater zidovudine *CL*_int_ value (Table 7); UGT1A9*1/2B7*2 dimer was as the most increased with an activity 460% that of UGT2B7*2 single expressed form. However, with only one exception, UGT1A9*3/2B7*2, all UGT2B7*N complexes with UGT1A9 variants (UGT1A9*2, *3, and *5) displayed a distinctly lower *CL*_int_ values, which ranged from ~20% to ~88% (Table 7).

## Discussion

Accumulating evidence demonstrates that human UGTs function in oligomeric forms^26^,^31^,^32^,^33^,^34^,^35^. Oligomerization of UGT1A subfamily members and UGT2B7 isoform, as well as their influences on metabolic activities, have continuously attracted interest. Recently, our lab reported homo- and hetero-dimerization of UGT2B7 allozymes^12^, as well as UGT1A1 and UGT1A9 allozymes^25^. In the current study, FRET and Co-IP were employed to determine inter-isoform interactions among UGT1A1, 1A9 and 2B7 enzymes, further establishing the relationship between protein interactions and alterations in catalytic properties using a panel of double expression systems.

FRET is a frequently used technique for assessing macromolecular interactions within 10 nm^27^,^36^. However, when the distance exceeds the valid amount (>10 nm), FRET is not capable of determining oligomerization, and Co-IP provides a complementary biochemical evidence for protein oligomerization. Employing quantitative FRET, we confirmed the existence of most hetero-dimers among UGT1A1, 1A9 and 2B7 allozymes. However, without significant FRET phenomena, the UGT1A1*1b and UGT2B7*5 dimer was finally detected by Co-IP, indicating that Co-IP might be more sensitive for identifying relatively weak interactions. FRET and Co-IP results obtained in this study revealed the promiscuous nature of UGT1A1, 1A9 and 2B7 allozymes to hetero-dimerize with each other, which is also supported by related literatures^23^,^24^. Compared with the homo-dimerization of UGT1A1, UGT1A9 and UGT2B7 allozymes^12^,^25^, inter-isoform hetero-dimerizing interactions were obviously weaker. This suggests that the homo-dimers of UGT1A1, UGT1A9 and UGT2B7 might be more predominant than hetero-dimers *in vivo*.

An array of reports have suggested the importance of oligomerization in biological functions of human UGTs^24^,^32^,^34^. In this study, the enzymatic features of target dimers were characterized using double expression systems. Distinct alterations of kinetics were observed compared to the corresponding single enzymes. Although the higher FRET efficiency (E%) and shorter donor-acceptor distance (*r*) indicate stronger interactions between the molecules in dimers, it is not directly correlated with the degree of influence on enzymatic activities. UGT1A1*1/2B7*1 dimer exhibited significantly higher E% and shorter *r* compared with UGT1A1*1/2B7*2 dimer (Table 1). However, compared with their corresponding single expressed enzymes, the latter hetero-dimerization caused more severe alterations of both quercetin (Fig. 4) and zidovudine (Fig. 9 and Table 6) glucuronidation activities.

Without the three-dimensional (3D) structures of UGT protein-protein complexes, it is difficult to predict the accurate interaction mechanism of the proteins. However, combination of quantitative FRET data and changes of kinetics parameters provide several hints for the dimerization mode. Based on calculated donor-acceptor distances and FRET efficiencies, the affinities of C-terminal truncated (absence of residues 445–530) mutant UGT1A1*1b to UGT1A9 and UGT2B7 allozymes were notably decreased compared with wild type UGT1A1. Nevertheless, UGT1A1*1b could still form dimers. These findings suggested that C-terminal residues 445–530 belong to the UGT1A1 protein interaction region. The constructed UGT1A1 3D-structural model by Laakkonen and Finel^37^ showed that residues 445–533 fold into two C-terminal envelop α-helixes (env1 and env2) and a transmembrane helix (TM-helix) (Fig. S14). UGT1A subfamily members share an identical C-terminal domain; thus, it is likely that the corresponding part of UGT1A9 (residues 442–530) is also involved in protein interactions (Fig. S14). In terms of varying affinities to certain target proteins, C3Y (*2), M33T (*3), and D256N (*5) mutations of UGT1A9, as well as A71S (*71S), H268Y (*2) and D398N (*5) mutations of UGT2B7 may locate in the interface of the two molecules and interrupt protein interactions. The constructed UGT1A9 structural model showed that residues C3, M33 and D256 are in the N-terminal domain, in the signal peptide, loop between the Nβ1 sheet and Nα1 helix, and loop between the Nα9 helix and Cα0, respectively (Fig. S14)^38^. UGT1A9 and UGT1A1 share a relatively high sequence identity (66%) at the N-terminus. Probably, the N-terminus of UGT1A enzymes facilitates hetero-dimerization. The crystal structure of the C-terminal domain of UGT2B7 clearly revealed that residue D398 resides at Cα5, which is directly exposed to the solvent^39^. By multiple sequence alignment with UGT1A1 and UGT1A9, A71 and H268 are both considered N-terminal residues. Taken together, we speculate that hetero-dimerization of UGT1A1, 1A9, and 2B7 occurs via both N- and C-terminal domains.

The changes of kinetics also support our hypothesis regarding the UGTs interaction mode. It is commonly assumed that conformational changes arising from protein interactions play a critical role in catalytic activity alterations. The *K*_m_ value reflects the affinity between substrate and enzyme. Analyzing the kinetic parameters obtained for single and double expressed UGT1A1, 1A9 and 2B7, we found that most *K*_m_ values were markedly changed by protein interactions. Therefore, hetero-dimerization induced conformational changes of the substrate binding site, resulting in altered *K*_m_ value. Based on constructed 3D-structure models of human UGTs and the crystal structure of UGT2B7 C-terminal domain^37^,^38^,^39^,^40^,^41^, we propose that the substrate-binding site is located at the variable N-terminal region, and is involved in protein-protein interactions. Representing the affinity of a substrate to the enzyme, the *K*_m_ value directly correlates with clearance efficiency, which is evaluated by *CL*_int_. However, in many hetero-dimers tested in this study, sharply reduced *K*_m_ values did not lead to increased *CL*_int_, e.g. UGT1A9*2/2B7*N dimers for the catalysis of M1 formation. Conversely, with lower substrate affinity to the enzyme (higher *K*_m_ values), clearance efficiency of the co-expressed enzymes was even higher, e.g. the UGT1A1*1/2B7*1 dimer for catalyzing zidovudine conversion. Based on these observations, we hypothesize that protein dimerization may also change the spatial arrangement of the co-factor’s binding site at the highly conserved C-terminus, and compensate the effects on substrate binding sites. Combined with the above findings, protein-protein interactions in UGT hetero-dimers might extensively include both N- and C-terminal domains of UGTs.

Integrated with *K*_m_ and *V*_max_, clearance efficiency (*CL*_int_) is a parameter reflecting the overall metabolic activity of UGT enzymes. With respect to *CL*_int_ values, heterodimerization with various allozymes resulted in various alterations of catalytic activities. It should be mentioned that association with the inactive mutant UGT1A1*1b mostly facilitated a dominant negative effect on the glucuronidation activity of UGT1A9 and 2B7. Binding with UGT1A1*1b similarly inhibited the catalytic activity of wild type UGT1A1^25^. We speculate that UGT1A1*1b may serve as a negative modulator for the entire human UGT family rather than just the UGT1A1 isoform. Previously, we reported that most of hetero-dimerizations between UGT1A9 allozymes decreased quercetin glucuronidation activity^25^. Conversely, with a few exceptions, interactions with UGT2B7 allozymes enhance UGT1A9 dependent quercetin metabolism. For UGT2B7 allozymes, interactions with wild type UGT1A1 or UGT1A9 significantly elevated zidovudine catalytic activity, corroborating previous findings^24^. In the present study, UGT2B7 allozymes complexes with UGT1A9 mutants all displayed reduced glucuronidation activities. However, as reported by Fujiwara *et al*., the dimer of wild type UGT2B7 with UGT1A9*2 mutant exhibits a drastically higher activity than the single expressed UGT2B7^24^, contrasting our data. Differential expression system and protein tags may contribute to this discrepancy. In addition, we previously reported that dimerization affects the regioselectivity of UGT1A9 enzymes^25^. This phenomenon also occurs in a number of UGT1A9*N/2B7*N dimers. Concerning *K*_m_ values, UGT1A9*2 and *5 both prefer the 7-glucuronidation site of quercetin, while UGT1A9*2 dimers with UGT2B7*1, *71 or *5, and UGT1A9*5 with UGT2B7*1, *71 or *2 change to 3-glucuronidation site. UGT1A9*3 possesses a higher affinity to the 3-glucuronidation site than the 7-glucuronidation site. However, its complexes with UGT2B7*1 and *71S display a preference to the 7-glucuronidation site. The alterations of regioselectivity also suggest the conformational changes of substrate binding site at the N-terminal domain. Considering the complicated catalytic behaviors of UGT mutants and dimers, the associations of structure and function need to be fully established. Moreover, UGTs are key enzymes in drug metabolism. The enzymes terminate the biological actions of their substrates and enhance excretion. It is well known that UGTs form dimers *in vivo*; however, how dimeric interactions affect UGT activity remains undefined. We established a recombinant system to assess the activity of target hetero-dimers of UGT1A1, 1A9 and 2B7. This enables us to study the enzymes without disturbance from other homologues, still in an *in vivo* system. The obtained catalytic parameters of the dimers may serve as a reference for a more accurate prediction of drug metabolism *in vivo* as well as determination of individual drug administration. In conclusion, utilizing quantitative FRET and Co-IP, the current study demonstrated the inter-isoform hetero-dimerization of UGT1A1, 1A9, and 2B7 allozymes. Enzymatic activities were altered by dimerization, to varying degrees. It is conceivable that protein dimeric interactions induce conformational changes and result in altered catalytic properties. The obtained results provide key information for the combined effects of polymorphisms and dimerization on UGT1A1, 1A9 and 2B7 catalytic activity *in vivo*, and allow in-depth understanding of human UGTs.

## Materials and Methods

### Materials

Cellfectin II reagent, pFastBac1 vector, *E. coli* DH10Bac cells, Sf900II SFM, and Gibco fetal bovine serum were purchased from Invitrogen (Carlsbad, CA, USA). *Spodoptera frugiperda* Sf9 insect cells were obtained from the China Center for Type Culture Collection (Wuhan, China). Rabbit anti-human UGT2B7 polyclonal antibodies were purchased from ProteinTech Group, Inc. (Chicago, USA). Rabbit anti-UGT1A Polyclonal antibodies were obtained Institute of Genetics and Developmental Biology Chinese Academy of Sciences (Beijing, China). Rabbit anti-human UGT2B7 polyclonal antibodies were purchased from ProteinTech Group, Inc. (Chicago, USA).

Zidovudine and quercetin (chemical purity >98.5%) were purchased from National Institute for Food and Drug Control (Beijing, China). Zidovudine *O*-glucuronide was purchased from Toronto Research Chemicals (Toronto, Canada). Uridine 5-diphosphoglucuronic acid (UDPGA), alamethicin, paraformaldehyde (PFA) and deoxycholic acid were purchased from Sigma Chemical Co. (St. Louis, MO, USA). Nonidet P-40 and Tween-20 were purchased from Amresco Inc. (Solon, OH, USA). Tris-HCl, NaCl, EDTA, MgCl_2_, perchloric acid and KH_2_PO_4_ were purchased from Sinopharm Chemical Regent Co. (Beijing, China). Chromatographic grade methanol was purchased from Tedia, Co. (Fairfield, OH, USA).

### Heterologous Expression of UGT1A1, 1A9, and 2B7 in Sf9 cells

The pFastBac1-UGT1A1*N-CFP (UGT1A1*1, UGT1A1*1b), pFastBac1-UGT1A9*N-*CFP*, pFastBac1-UGT1A9*N-*YFP* and pFastBac1-UGT1A9*N-HA (UGT1A9*1, UGT1A9*2, UGT1A9*3, and UGT1A9*5), pFastBac1-UGT2B7*N-YFP and pFastBac1-UGT2B7*N-HA (UGT2B7*1, UGT2B7*71S, UGT2B7*2, and UGT2B7*5) plasmids were previously constructed. Heterologous expressions of UGT1A1 1A9, and 2B7 with Bac-to-Bac system were carried out as the method described by our lab^25^. After transformation of recombinant plasmids into the competent *E. coli* DH10Bac cells, recombinant bacmid-UGT1A1*N-CFP, bacmid-UGT1A9*N-CFP, bacmid-UGT1A9*N-YFP, bacmid-UGT1A9*N-HA bacmid-UGT2B7*N-YFP and bacmid-UGT2B7*N-HA were separately purified and confirmed. Subsequently, Sf9 cells were transfected with recombinant bacmids and cultured at 27 °C in Sf900II SFM medium. The first passage *baculovirus* was collected after 72 h of transfection. For protein production, a high titer of recombinant *baculovirus* was obtained by repeating the amplification.

### FRET analysis

Sf9 cells with a density of 2 × 10^6^ cells/well were seeded on 6-well plates and co-infected with recombinant UGT1A1*N-CFP/1A9*N-YFP, UGT1A1*N-CFP/2B7*N-YFP, UGT1A9*N-CFP/2B7*N-YFP baculovirus. Separately infected with UGT1A1*N-CFP, UGT1A9*N-CFP, UGT1A9*N-YFP, UGT2B7*N-YFP baculovirus, the cells were applied for the fluorescence detection of single expressed enzymes. Cells co-infected with *CFP* and *YFP* baculovirus were used as the negative control, while *CFP-linker-YFP* treated cells served as the positive control. After 72 h of infection, the cells were fixed using 4% PFA for 30 min at 4 °C. Fixed cells were washed with phosphate buffered saline (PBS) buffer and mounted on slides. The FRET was measured with an Olympus BX61W1-FV1000 confocal microscope (Olympus, Tokyo, Japan). The acceptor photobleaching method was employed for the determination of FRET efficiency^42^. Images of fixed Sf9 cells were taken at both CFP (excitation: 405 nm, emitter: 476 nm) and YFP (excitation: 515 nm, emitter: 527 nm) channels. Following was the bleaching procedure. Avoiding detectable photobleaching, the laser intensity was adjusted to less than 5% of the laser power at both of CFP (405 nm) and YFP (515 nm) channels. To achieve the best possible dynamic range, the crosstalk between CFP and YFP channel was eliminated by adjusting the gain of the photomultiplier tube (PMT). Lastly, a selected Sf9 cell was bleached at 515 nm with 100% laser intensity. Two images before photobleaching and eight images after photobleaching were collected at both CFP and YFP channels. The collected data were processed by OLYMPUS FLUOVIEW FV1000 software. The equation *E* = (*D*_*a*_ − *D*_*b*_)*/D*_*a*_ was used for the calculation of FRET efficiency (*E*), where *D*_*a*_ and *D*_*b*_ are the donor fluorescence intensities after and before photobleaching, respectively. The distance (*r*) between the donor and acceptor was determined by the equation *E* = *R*_*0*_^6^*/ (R*_*0*_^*6*^ + *r*^*6*^), where *R*_*0*_ is the forster distance (52.16 Å, manufacture data).

### Co-immunoprecipitation

Sf9 cells with a density of 2 × 10^6^ cells/well were seeded and co-infected with recombinant UGT1A1*N-CFP/1A9*N-HA, UGT1A1*N-CFP/2B7*N-HA, and UGT1A9*N-CFP/2B7*N-HA baculovirus. The cells were collected after 72 h, and lysed with 1 mL lysis buffer (0.05 M Tris-HCl, pH 7.4, 0.15 M NaCl, 0.25% deoxycholic acid, 1% Nonidet P-40, 1 mM EDTA) containing 1% protease inhibitor cocktail (Sigma Chemical Co., USA). After centrifugation, the supernatant was mixed with 30 μL of anti-HA beads (Roche Applied Science, Germany) and incubated overnight at 4 °C. After washing with cold lysis buffer, anti-HA beads were loaded on SDS-PAGE followed by Western blot analysis with anti-UGT1A and anti-UGT2B7 antibodies.

### Western blot analysis

After separation by SDS-PAGE, co-immunoprecipitation samples were transferred onto a polyvinylidene fluoride (PVDF) membrane (Millipore, Bedford, MA). PBST (1 × PBS, 0.1% Tween-20) containing 10% nonfat milk was used for membrane blocking. Subsequently, the PVDF membrane was incubated with rabbit anti-human UGT1A and UGT2B7 polyclonal antibodies diluted 1:2000 for 2 h at room temperature. Horseradish peroxidase conjugated goat anti-rabbit secondary antibodies diluted 1:5000 were then added for 1 h at room temperature. After PBST wash, the membrane was visualized using SuperSignal West Pico (Pierce) and exposed on an X-ray film (Kodak).

For assessing the relative expression levels of CFP-tagged UGT1A1 and UGT1A9, and HA-tagged UGT1A9 and UGT2B7 allozymes in single or double expression systems, total cell homogenates (0.8–16 μg) were separated by 10% SDS-PAGE and transferred onto a nitrocellulose membrane. Western blot analysis was carried out with anti-UGT1A and anti-UGT2B7 antibodies; band intensities were analyzed on an Odyssey infrared imaging system (LI-COR Biosciences). UGT1A1*1^HA^ and UGT2B7*1^HA^ single expression levels were set as 100% for the standard curve. By comparing Western blot densities to those of the calibration curve, the relative expression levels of UGT1A1*N^CFP^, UGT1A9*N^CFP^, UGT1A9*N^HA^ and UGT2B7*N^HA^ allozymes were quantified and used for kinetic experiments.

### Enzyme activity assay

For determination of relative quercetin glucuronidation activities of UGT1A1*N^CFP^/1A9*N^HA^ and UGT1A1*N^CFP^ /2B7*N^HA^ double expression systems, 1 mg/mL total cell homogenates, 0.1 mM quercetin, and alamethicin (50 mg/mg of protein) were incubated in 100 μL Tris-HCl buffer (0.1 M, pH = 7.4). After 5 min of pre-incubation, the reaction was initiated by adding UDPGA at 37 °C, and was terminated by adding 300 μL leuteolin in 30 min. Following centrifugation (13,000 × g, 10 min), the supernatant was subjected into high performance liquid chromatography (HPLC) system. Kinetic parameters of quercetin conversion by UGT1A1*N^CFP^/1A9*N^HA^ and UGT1A9*N^CFP^/2B7*N^HA^ double expression systems were measured with a serial concentrations of quercetin (5.011–150.330 μM). A total volume of 100 μL potassium phosphate buffer (50 mM, pH 7.4) containing 1.5 mg/mL total cell homogenates, 5 mM MgCl_2_, 10 mM UDPGA, alamethicin (50 mg/mg of protein), and zidovudine (75.12–2403.84 mM) was used for zidovudine glucuronidation assay of the co-expressed UGT2B7*N^HA^ with UGT1A1*N^CFP^ or UGT1A9*N^CFP^. The assay system was pre-incubated for 5 min. As the formation of zidovudine *O*-glucuronide was linear with time-dependent increase within 120 min, the reaction time was set as 120 min at 37 °C. Adding 10 mL of 30% perchloric acid, the mixture was centrifugated at 13,000 × g for 10 min. The supernatant was analyzed by HPLC. An Agilent 1200 system, equipped with an Agilent ExtendTM C18 (250 mm × 4.6 mm, 5 μm) column, was used for the analysis of glucuronidation. The mobile phase for separation of quercetin and its metabolites consisted of methanol and 0.02 M pH 2.0 phosphoric acid (46:54, v/v) with a flow rate of 1 mL/min. The detection wavelength was 368 nm. Zidovudine and its glucuronide were analyzed under the following conditions: 16% methanol and 84% KH_2_PO_4_ (10 mM, pH 2.2), 1.0 mL/min flow, and 265 nm detection wavelength.

### Kinetic analyses of glucuronidation

The software GraphPad Prism, version 5.0 (GraphPad Software Inc., San Diego, USA) was employed for kinetic analysis. The equations, including Michaelis-Menten equation, *v = V*_max_ · *S*/(*K*_m_ + *S*), the substrate inhibition model, *v = V*_max_/(1 + (*K*_m_/*S*) + (*S*/*K*_si_), and *CL*_int_ (intrinsic clearance)* = V*_max_/*K*_m_, were used for the kinetic data calculation, where *v* is the velocity of the reaction, *V*_max_ is the maximum velocity, *S* is the substrate concentration, *K*_m_ is the Michaelis-Menten constant, *K*_si_ is the constant describing the substrate inhibition interaction.

### Statistical analysis

The means ± standard deviation (SD) were obtained from triplicate experiments. One-way analysis of variance (ANOVA) and Dunnett’s post-hoc test (SPSS 13.0 software, SPSS Inc., Chicago, IL, USA) were utilized for determination of significance of differences. *P* < 0.05 was considered statistically significant.

## Additional Information

**How to cite this article**: Yuan, L.-M. *et al*. Inter-isoform Hetero-dimerization of Human UDP-Glucuronosyltransferases (UGTs) 1A1, 1A9, and 2B7 and Impacts on Glucuronidation Activity. *Sci. Rep.* **6**, 34450; doi: 10.1038/srep34450 (2016).

**Publisher’s note**: Springer Nature remains neutral with regard to jurisdictional claims in published maps and institutional affiliations.

## Supplementary Material

Supplementary Information

## Figures and Tables

**Table 1:** FRET efficiency (E%) and donor-acceptor distances *r.*

	FRET efficiency E %	Donor-acceptor distance *r* (nm)	*n*
CFP/YFP	−1.56 ± 4.8	>10	5
CFP-linker-YFP	17.4 ± 4.8	6.92	21
1A1*1^CFP/^1A9*1^YFP^	18.7 ± 8.2	6.74	14
1A1*1^CFP/^1A9*2^YFP^	12.3 ± 4.1	7.32	15
1A1*1^CFP/^1A9*3^YFP^	24.3 ± 8.3	6.38	9
1A1*1^CFP/^1A9*5^YFP^	10.3 ± 7.4	7.57	18
1A1*1^CFP^/2B7*1^YFP^	20.7 ± 6.9	6.60	15
1A1*1^CFP^/2B7*71S^YFP^	9.94 ± 4.9	7.62	13
1A1 *1^CFP^/2B7*2^YFP^	6.41 ± 2.4	8.25	10
1A1 *1^CFP^/2B7*5^YFP^	9.38 ± 7.7	7.70	23
1A1*1b^CFP/^1A9*1^YFP^	9.29 ± 5.5	7.71	25
1A1*1b^CFP/^1A9*2^YFP^	5.33 ± 3.7	8.52	19
1A1*1b^CFP/^1A9*3^YFP^	6.51 ± 3.6	8.23	23
1A1*1b^CFP/^1A9*5^YFP^	8.10 ± 3.9	7.91	10
1A1*1b^CFP^/2B7*1^YFP^	8.00 ± 4.7	7.93	17
1A1*1b^CFP^/2B7 *71S^YFP^	3.19 ± 4.4	9.32	20
1A1*1b^CFP^/2B7*2^YFP^	5.36 ± 3.6	8.51	11
1A1*1b^CFP^/2B7*5^YFP^	−0.74 ± 2.9	>10	10
1A9*1^CFP^/2B7 *1^YFP^	17.9 ± 7.3	6.80	16
1A9*1^CFP^/2B7*71S^YFP^	11.8 ± 7.5	7.38	12
1A9*1^CFP^/2B7*2^YFP^	6.57 ± 2.6	8.21	12
1A9*1^CFP^/2B7*5^YFP^	9.53 ± 5.7	7.68	11
1A9*2^CFP^/2B7*1^YFP^	10.3 ± 3.7	7.57	8
1A9*2^CFP^/2B7*71S^YFP^	9.23 ± 5.5	7.72	25
1A9*2 ^CFP^/2B7*2^YFP^	12.3 ± 2.1	7.32	13
1A9*2^CFP^/2B7*5^YFP^	3.81 ± 4.1	9.04	15
1A9*3^CFP^/2B7*1^YFP^	9.03 ± 5.8	7.75	14
1A9*3^CFP^/2B7*71S^YFP^	9.31 ± 5.4	7.71	10
1A9*3^CFP^/2B7*2^YFP^	4.76 ± 2.9	8.69	10
1A9*3^CFP^/2B7*5^YFP^	12.2 ± 4.3	7.33	16
1A9*5^CFP^/2B7*1^YFP^	11.4 ± 6.8	7.42	13
1A9*5^CFP^/2B7*71S^YFP^	7.71 ± 4.4	7.98	26
1A9*5^CFP^/2B7*2^YFP^	4.88 ± 2.3	8.66	17
1A9*5^CFP^/2B7*5^YFP^	13.4 ± 7.2	7.20	17

*n* is the sample size.

**Table 2:** Kinetic parameters of M1 (quercetin-7-glucuronide) formation by single expressed UGT1A9*N^HA^ and double expressed UGT1A1*1b^CFP^/1A9*N^HA^ systems.

1A9*N or 1A1*1b/1A9*N	*K*_m_ (μM)	*K*_si_ (μM)	*V*_max_ (pmol/min/unit)	*CL*_int_ (μL/min/unit)	% of 1A9*N^HA^
1A9*1^HA^	9.10 ± 0.21	1.56 ± 0.06	791.3 ± 4.160	45.30	100
1A1*1b^CFP^/1A9*1^HA^	14.87 ± 0.43***	1.93 ± 0.08	90.55 ± 0.47***	3.05***	6.7
1A9*2^HA^	12.62 ± 1.97		257.6 ± 9.50	20.41	100
1A1*1b^CFP^/1A9*2^HA^	11.11 ± 1.84		45.93 ± 1.85***	4.13***	20.2
1A9*3^HA^	42.34 ± 8.23		768.2 ± 55.91	18.14	100
1A1*1b^CFP^/1A9*3^HA^	41.36 ± 7.71		328.8 ± 22.25**	7.95**	43.8
1A9*5^HA^	12.40 ± 1.43		68.02 ± 1.98	5.48	100
1A1*1b^CFP^/1A9*5^HA^	7.39 ± 1.16*		16.87 ± 0.57***	2.28***	41.6

Asterisks indicate statistically significant differences compared with UGT1A9*N^HA^ single expression systems (***P < 0.0001, **P < 0.005, *P < 0.05). Data are mean ± SD of three independent determinations.

**Table 3:** Kinetic parameters of M2 (quercetin-3-glucuronide) formation by single expressed UGT1A9*N^HA^ and double expressed UGT1A1*1b^CFP^/1A9*N^HA^ systems.

1A9*N or 1A1*1b/1A9*N	*K*_m_ (μM)	*K*_si_ (μM)	*V*_max_ (pmol/min/unit)	*CL*_int_ (μL/min/unit)	% of 1A9*N^HA^
1A9*1^HA^	7.74 ± 0.86		568.0 ± 13.30	37.08	100
1A1*1b^CFP^/1A9*1^HA^	10.29 ± 0.98*	256.9 ± 36.17	135.9 ± 5.73***	13.21***	35.6
1A9*2^HA^	3.65 ± 0.40		211.0 ± 3.54	57.89	100
1A1*1b^CFP^/1A9*2^HA^	4.02 ± 0.70	117.5 ± 16.94	88.35 ± 5.09*	22.00***	38.0
1A9*3^HA^	21.85 ± 4.80	195.7 ± 61.83	467.3 ± 57.26	21.39	100
1A1*1b^CFP^/1A9*3^HA^	14.65 ± 2.77	147.0 ± 34.84	664.9 ± 64.76*	45.39**	212.2
1A9*5^HA^	7.21 ± 1.39		34.75 ± 1.43	4.82	100
1A1*1b^CFP^1A9*5^HA^	4.13 ± 1.15*	103.3 ± 23.16	30.73 ± 2.91	7.44**	154.4

Asterisks indicate statistically significant differences compared with UGT1A9*N^HA^ single expression systems (***P < 0.0001, **P < 0.005, *P < 0.05). Data are mean ± SD of three independent determinations.

**Table 4:** Kinetic parameters of M1 (quercetin-7-glucuronide) formation by single expressed UGT1A9*N^CFP^ and double expressed UGT1A9*N^CFP^/2B7*N^HA^ systems.

1A9*N or 1A9*N/2B7*N	*K*_m_ (μM)	*V*_max_ (pmol/min/unit)	*CL*_int_ (μL/min/unit)	% of 1A9*N^CFP^
1A9*1^CFP^	14.99 ± 0.88	1868 ± 35.67	69.43	100
1A9*1^CFP^/2B7*1^HA^	10.64 ± 1.86*	4469 ± 301.4***	258.7***	372.6
1A9*1^CFP^/2B7*71S^HA^	10.03 ± 0.63**	2567 ± 31.33***	130.4***	187.8
1A9*1^CFP^/2B7*2^HA^	11.20 ± 1.75*	4505 ± 159.9***	402.2***	579.3
1A9*1^CFP^/2B7*5^HA^	9.82 ± 0.93**	2334 ± 48.23**	237.7***	342.4
1A9*2^CFP^	15.58 ± 1.82	263.7 ± 8.304	16.93	100
1A9*2^CFP^/2B7*1^HA^	9.82 ± 1.72*	164.1 ± 4.60***	16.71	98.7
1A9*2^CFP^/2B7*71S^HA^	8.99 ± 0.99*	275.3 ± 6.90	30.62***	180.9
1A9*2^CFP^/2B7*2^HA^	9.00 ± 0.95*	186.1 ± 4.50**	20.67**	122.1
1A9*2^CFP^/2B7*5^HA^	6.43 ± 0.79**	76.13 ± 1.92***	11.84**	69.9
1A9*3^CFP^	48.11 ± 6.05	600.7 ± 29.61	12.49	100
1A9*3^CFP^/2B7*1^HA^	28.72 ± 1.97	1029 ± 27.56***	19.36***	155.0
1A9*3^CFP^/2B7*71S^HA^	28.97 ± 11.27	553.5 ± 63.25	19.11*	153.0
1A9*3^CFP^/2B7*2^HA^	35.30 ± 5.91	1068 ± 64.32**	30.25***	242.2
1A9*3^CFP^/2B7*5^HA^	25.24 ± 2.74	746.6 ± 31.95**	16.59**	132.8
1A9*5^CFP^	22.41 ± 1.99	408.7 ± 10.99	18.24	100
1A9*5^CFP^/2B7*1^HA^	13.56 ± 1.35**	146.5 ± 3.78***	10.80***	59.2
1A9*5^CFP^/2B7*71S^HA^	15.56 ± 1.31*	480.9 ± 10.96**	30.91***	169.5
1A9*5^CFP^/2B7*2^HA^	11.19 ± 1.29**	316.7 ± 8.91**	28.30***	155.2
1A9*5^CFP^/2B7*5^HA^	26.30 ± 5.25	263.2 ± 16.72**	10.01***	54.9

Asterisks indicate statistically significant differences compared with UGT1A9*N^CFP^ single expression systems (***P < 0.0001, **P < 0.005, *P < 0.05). Data are mean ± SD of three independent determinations.

**Table 5:** Kinetic parameters of M2 (quercetin-3-glucuronide) formation by single expressed UGT1A9*N^CFP^ and double expressed UGT1A9*N^CFP^/2B7*N^HA^ systems.

1A9*N^CFP^ or 1A9*N/2B7*N	*K*_m_ (μM)	*K*_si_ (μM)	*V*_max_ (pmol/min/unit)	*CL*_int_ (μL/min/unit)	% of 1A9*N^CFP^
1A9*1^CFP^	7.79 ± 0.34		1575 ± 8.08	109.7	100
1A9*1^CFP^/2B7*1^HA^	7.78 ± 1.09		5102 ± 203.1***	357.0***	325.4
1A9*1^CFP^/2B7*71S^HA^	8.16 ± 1.48*		2861 ± 158.9***	196.7***	179.3
1A9*1^CFP^/2B7*2^HA^	8.22 ± 0.59		4175 ± 67.87***	274.3***	250.1
1A9*1^CFP^/2B7*5^HA^	2.57 ± 0.25		2694 ± 33.12***	1049***	956.8
1A9*2^CFP^	21.02 ± 6.99	233.0 ± 120.4	175.5 ± 31.82	8.35	100
1A9*2^CFP^/2B7*1^HA^	8.17 ± 0.65*		194.7 ± 4.05	12.33*	147.6
1A9*2^CFP^/2B7*71S^HA^	6.81 ± 1.16*		372.0 ± 24.75**	29.59***	354.5
1A9*2^CFP^/2B7*2^HA^	7.29 ± 1.59*	251.2 ± 74.53	300.3 ± 25.82*	41.22***	493.7
1A9*2^CFP^/2B7*5^HA^	12.75 ± 2.21	209.2 ± 50.92	151.1 ± 12.60	11.85*	141.9
1A9*3^CFP^	34.39 ± 7.55	139.5 ± 40.24	688.3 ± 96.12	20.01	100
1A9*3^CFP^/2B7*1^HA^	77.71 ± 34.34	105.2 ± 59.67	1572 ± 524.9*	20.23	101.0
1A9*3^CFP^/2B7*71S^HA^	39.23 ± 11.71	85.86 ± 29.50	788.0 ± 159.7	20.09	100.4
1A9*3^CFP^/2B7*2^HA^	7.66 ± 1.60		485.4 ± 22.87*	63.37**	316.6
1A9*3^CFP^/2B7*5^HA^	6.14 ± 0.52**		378.1 ± 5.00*	30.94**	154.6
1A9*5^CFP^	34.65 ± 9.19	211.5 ± 87.71	448.7 ± 73.77	12.95	100
1A9*5^CFP^/2B7*1^HA^	7.13 ± 0.41		137.9 ± 0.98**	9.83***	75.9
1A9*5^CFP^/2B7*71S^HA^	6.50 ± 0.42*		464.5 ± 3.40	35.97***	277.8
1A9*5^CFP^/2B7*2^HA^	14.17 ± 3.00*	286.4 ± 99.91	415.5 ± 42.54**	29.32**	226.4
1A9*5^CFP^/2B7*5^HA^	9.56 ± 2.79*		308.8 ± 36.08*	20.81*	160.7

Asterisks indicate statistically significant differences compared with UGT1A9*N^CFP^ single expression systems (***P < 0.0001, **P < 0.005, *P < 0.05). Data are mean ± SD of three independent determinations.

**Table 6:** Kinetic parameters of zidovudine *O*-glucuronidation by single expressed UGT2B7*N^HA^ and double expressed UGT1A1*N^CFP^/2B7*N^HA^ systems.

2B7*N or 1A1*N/2B7*N	*K*_m_ (μM)	*V*_max_ (pmol/min/unit)	*CL*_int_ (nL/min/unit)	% of 2B7*N^HA^
2B7*1^HA^	508.2 ± 18.59	37.64 ± 0.50	74.06	100
1A1*1^CFP^/2B7*1^HA^	585.2 ± 17.52*	66.47 ± 0.76***	113.59***	153.4
1A1*1b^CFP^/2B7*1^HA^	490.3 ± 43.26	40.55 ± 1.26*	82.70*	111.7
2B7*71S^HA^	533.5 ± 25.61	68.80 ± 1.23	141.86	100
1A1*1^CFP^/2B7*71S^HA^	456.4 ± 13.43*	49.76 ± 0.51***	109.03**	84.6
1A1*1b^CFP^/2B7*71S^HA^	806.0 ± 62.58**	41.17 ± 1.38***	51.08***	39.62
2B7*2^HA^	570.1 ± 34.18	24.88 ± 0.57	48.01	100
1A1*1^CFP^/2B7*2^HA^	395.2 ± 12.61**	72.91 ± 0.77***	184.49***	422.6
1A1*1b^CFP^/2B7*2^HA^	621.9 ± 34.19	17.64 ± 0.38***	28.36***	59.1

Asterisks indicate statistically significant differences compared with UGT2B7*N^HA^ single expression systems (***P < 0.0001, **P < 0.005, *P < 0.05). Data are mean ± SD of three independent determinations.

**Table 7:** Kinetic parameters of zidovudine *O*-glucuronidation by single expressed UGT2B7*N^HA^ and double expressed UGT1A9*N^CFP^/2B7*N^HA^ systems.

2B7*N or 1A9*N/2B7*N	*K*_m_ (μM)	*V*_max_ (pmol/min/unit)	*CL*_int_ (nL/min/unit)	% of 2B7*N^HA^
2B7*1^HA^	508.2 ± 18.59	37.64 ± 0.50	74.06	100
1A9*1^CFP^/2B7*1^HA^	469.1 ± 16.85	75.96 ± 0.97***	161.93***	218.6
1A9*2^CFP^/2B7*1^HA^	638.0 ± 26.27**	27.79 ± 0.45***	43.56***	58.8
1A9*3^CFP^/2B7*1^HA^	539.8 ± 25.84	35.30 ± 0.63*	66.01**	88.3
1A9*5^CFP^/2B7*1^HA^	606.4 ± 34.71*	17.74 ± 0.39***	29.25***	39.5
2B7*71S ^HA^	533.5 ± 25.61	68.80 ± 1.23	128.96	100
1A9*1^CFP^/2B7*71S ^HA^	502.7 ± 30.23	103.7 ± 2.27***	206.29***	160.0
1A9*2^CFP^/2B7*71S ^HA^	751.3 ± 33.11**	50.38 ± 0.95***	67.06***	52.0
1A9*3^CFP^/2B7*71S 1^HA^	626.8 ± 26.30*	37.94 ± 0.63***	60.53***	38.2
1A9*5^CFP^/2B7*71S ^HA^	502.4 ± 28.33	15.83 ± 0.32***	31.51***	19.9
2B7*2^HA^	570.1 ± 34.18	24.88 ± 0.57	43.64	100
1A9*1^CFP^/2B7*2^HA^	381.1 ± 17.54**	76.63 ± 1.16***	201.08***	460.7
1A9*2^CFP^/2B7*2^HA^	588.7 ± 25.32	15.04 ± 0.25***	25.55***	58.5
1A9*3^CFP^/2B7*2^HA^	674.8 ± 31.06*	32.26 ± 0.60***	47.81*	109.5
1A9*5^CFP^/2B7*2^HA^	783.8 ± 28.45**	9.566 ± 0.15***	12.20***	28.0

Asterisks indicate statistically significant differences compared with UGT2B7*N^HA^ single expression systems (***P < 0.0001, **P < 0.005, *P < 0.05). Data are mean ± SD of three independent determinations.
