# Supplementary material for: Inter-isoform Hetero-dimerization of Human UDP-Glucuronosyltransferases (UGTs) 1A1, 1A9, and 2B7 and Impacts on Glucuronidation Activity
Source: Sci Rep. 2016 Nov 18;6:34450. doi: 10.1038/srep34450 (PMC5114717; doi:10.1038/srep34450)
Supplement: Supplementary Information [file srep34450-s1.pdf]

Supporting Information

Inter-isoform Hetero-dimerization of Human UDP-Glucuronosyltransferases (UGTs) 1A1, 1A9, and 2B7 and Impacts on Glucuronidation Activity

Ling-Min Yuan[+], Zhang-Zhao Gao[+], Hong-Ying Sun, Sai-Nan Qian, Yong-Sheng Xiao, Lian-Li Sun* and Su Zeng*

Institute of Drug Metabolism and Pharmaceutical Analysis, Zhejiang Province Key Laboratory of Anti-Cancer Drug Research, College of Pharmaceutical Sciences, Zhejiang University, Hangzhou, 310058, China

*Corresponding author: Lianli Sun, Associate Professor. Address: College of Pharmaceutical Sciences, Zhejiang University, Hangzhou, China. Tel: +86 571 88208407; Fax: +86 571 88208407; E-mail: sunlianli@zju.edu.cn; Su Zeng, Professor. Address: College of Pharmaceutical Sciences, Zhejiang University, Hangzhou, China. Tel: +86 571 88208405; Fax: +86 571 88208405; E-mail: zengsu@zju.edu.cn

[+]These authors contributed equally to this work.

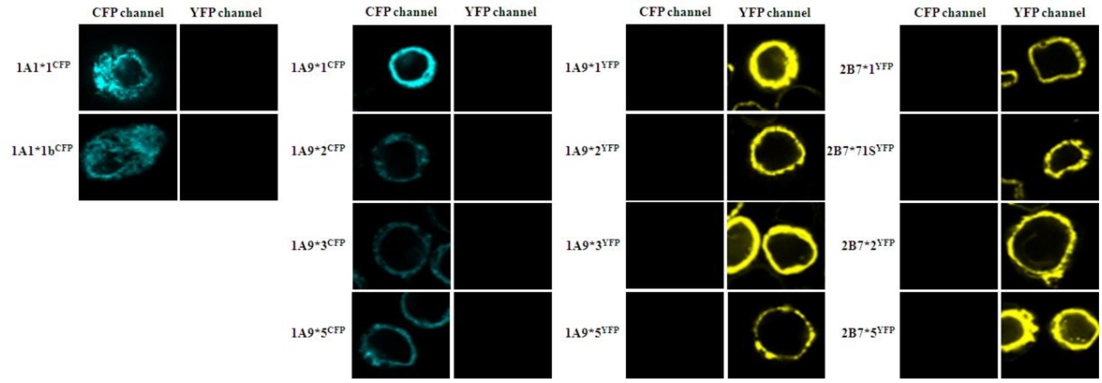

Figure S1. Fluorescence detection of UGT1A1*N^{CFP}, UGT1A9*N^{CFP}, UGT1A9*N^{YFP} and UGT2B*N^{YFP}.

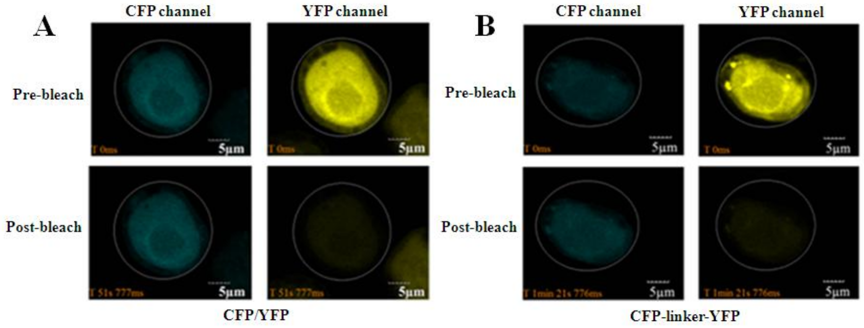

Figure S2. FRET detection of negative and positive controls using the acceptor photobleaching method. (A) Negative control: Sf9 cells co-infected with recombinant *CFP* and *YFP* baculovirus. Bleached by 515 nm laser line, CFP fluorescence of the circled area was not increased. (B) Positive control: Sf9 cells infected with *CFP-linker-YFP* baculouvirus. Bleached by 515 nm laser line, CFP fluorescence of the circled area was increased.

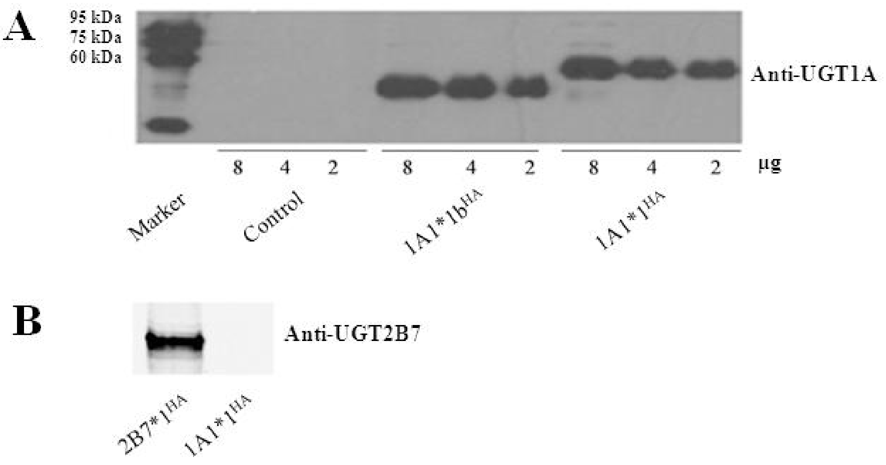

Figure S3. Western blot analysis of UGT1A1*1, 1A1*1b and 2B7*1 in the single expression system. Anti-UGT1A antibodies and anti-UGT2B7 antibodies specifically recognized UGT1A1 and 2B7, respectively. Cell lysates from Sf9 without baculovirus transfection were used as control.

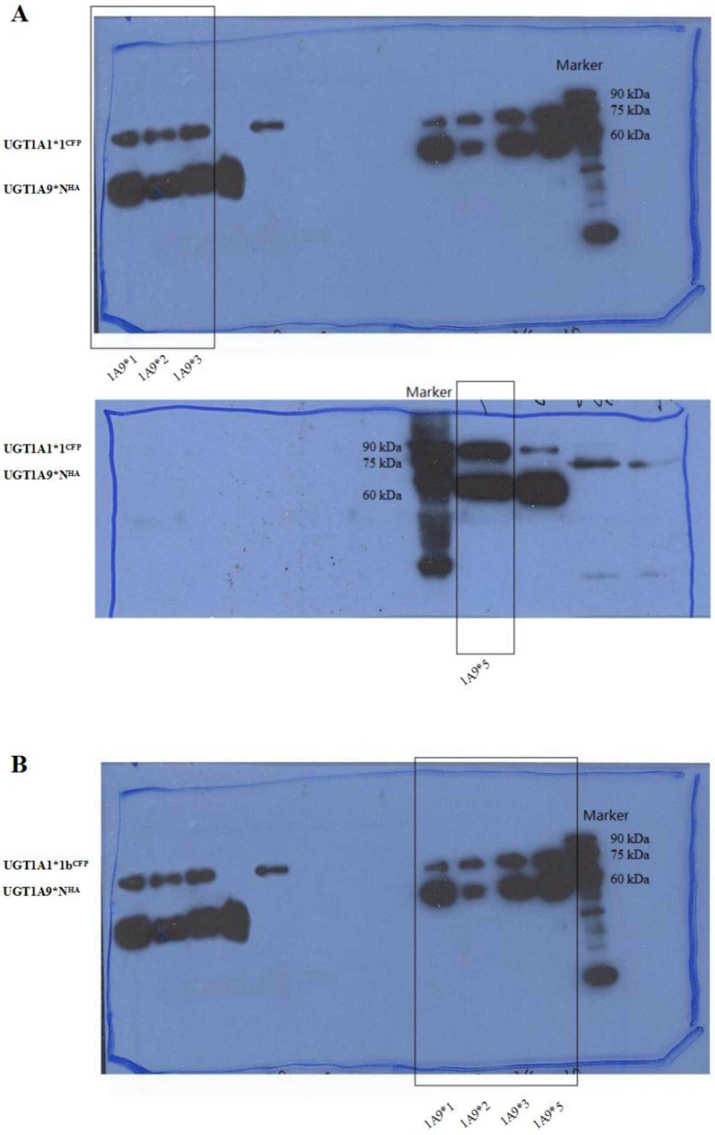
A
Marker
90 kDa
75 kDa
60 kDa
UGT1A1*1CFP
UGT1A9*NHA
1A9*1
1A9*2
1A9*3
Marker
UGT1A1*1CFP
UGT1A9*NHA
90 kDa
75 kDa
60 kDa
1A9*5
B
Marker
90 kDa
75 kDa
60 kDa
UGT1A1*1bCFP
UGT1A9*NHA
1A9*1
1A9*2
1A9*3
1A9*5

C

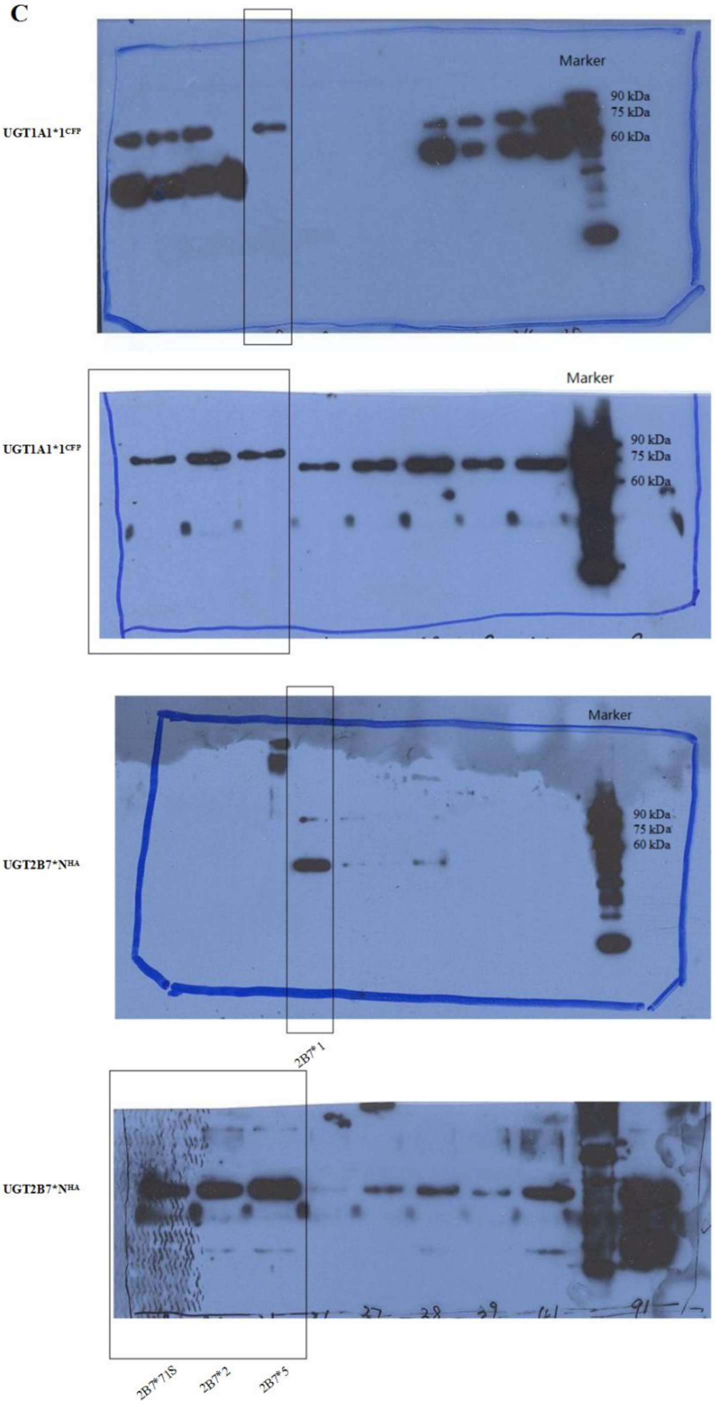
UGT1A1*1CFP
Marker
90 kDa
75 kDa
60 kDa
UGT1A1*1CFP
Marker
90 kDa
75 kDa
60 kDa
UGT2B7*NHA
Marker
90 kDa
75 kDa
60 kDa
2B7*1
UGT2B7*NHA
2B7*71S
2B7*2
2B7*5

D

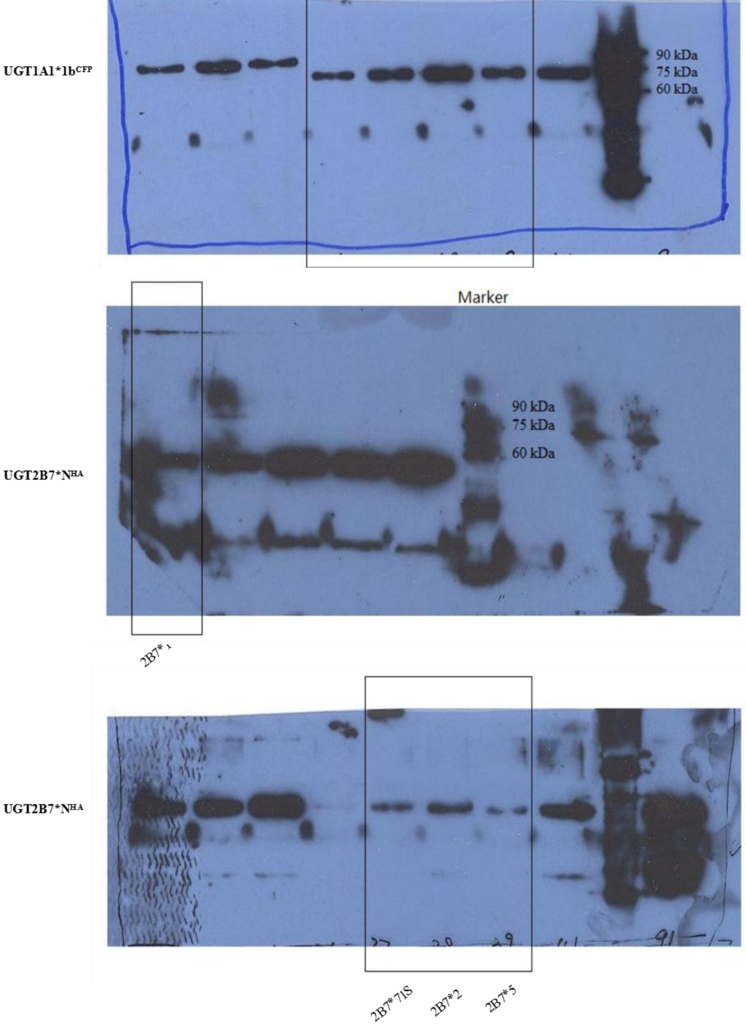
Marker
UGT1A1*1b^CFP
90 kDa
75 kDa
60 kDa
Marker
UGT2B7*N^HA
90 kDa
75 kDa
60 kDa
2B7*1
UGT2B7*N^HA
2B7*71S
2B7*2
2B7*5

E

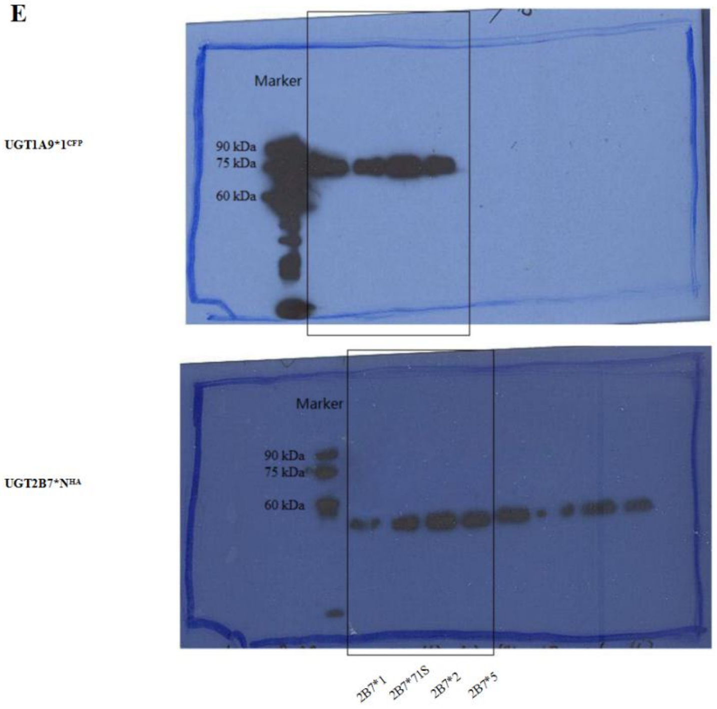
Marker
UGT1A9*1^CFP
90 kDa
75 kDa
60 kDa
Marker
UGT2B7*N^HA
90 kDa
75 kDa
60 kDa
2B7*1
2B7*71S
2B7*2
2B7*5

F

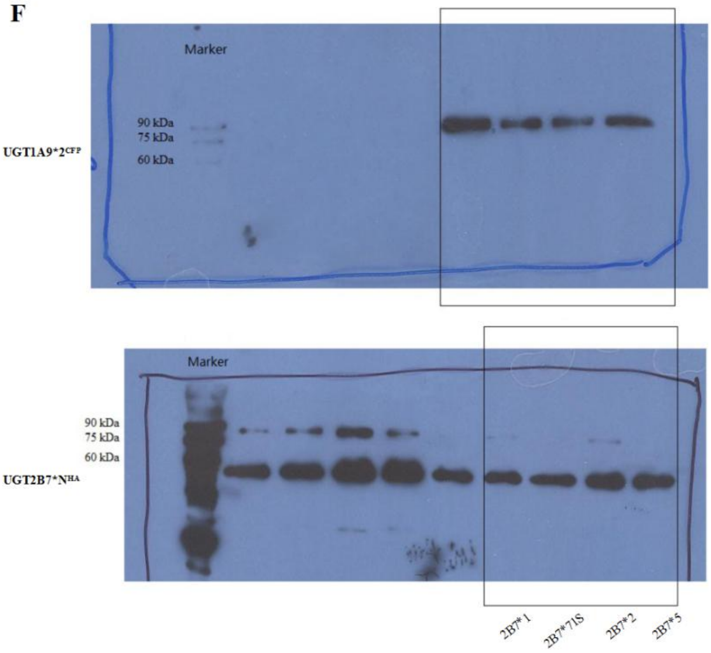
Marker
90 kDa
75 kDa
60 kDa
UGT1A9*2CFP
Marker
90 kDa
75 kDa
60 kDa
UGT2B7*NHA
2B7*1
2B7*71S
2B7*2
2B7*5

G

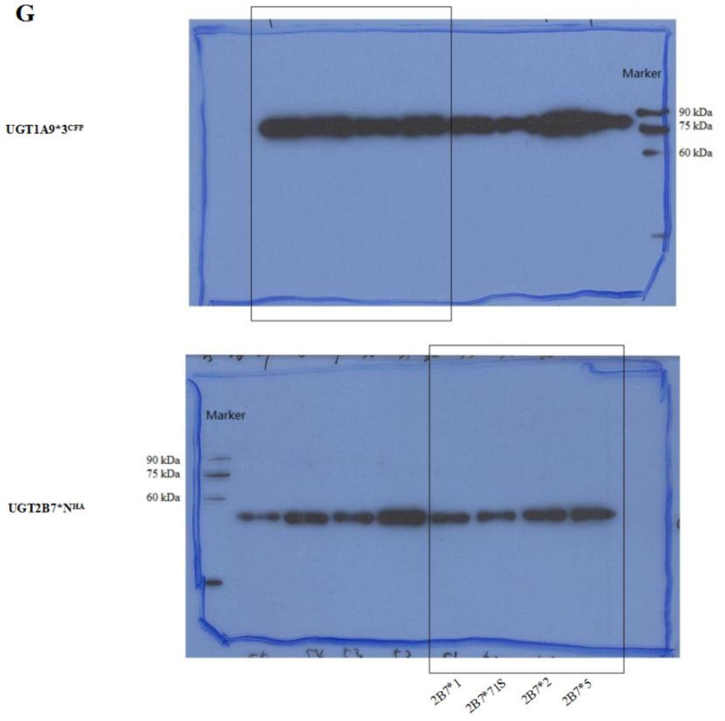

H

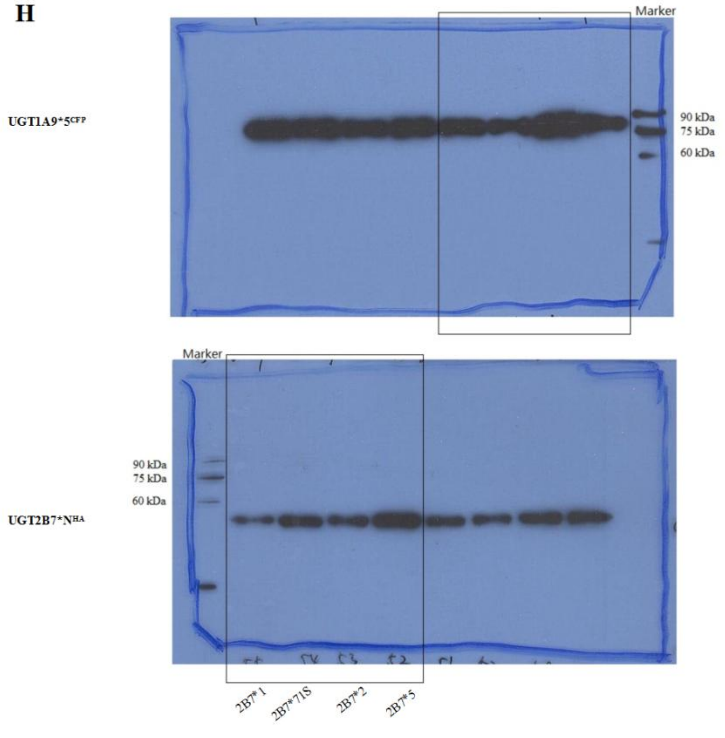

Figure S4. Co-IP analysis of cell lysates from UGT1A1, 1A9, and 2B7 double expression systems. Sf9 cells were co-infected with UGT1A1*N^{CFP}/1A9*N^{HA}, UGT1A1*N^{CFP}/2B7*N^{HA}, or UGT1A9*N^{CFP}/2B7*N^{HA} baculovirus. Cell lysates from double expression systems were

immunoprecipitated with anti-HA beads followed by Western blot analysis using anti-UGT1A and anti-UGT2B7 antibodies. Images A to H represent full-length Western blot results corresponding to Fig. 2.

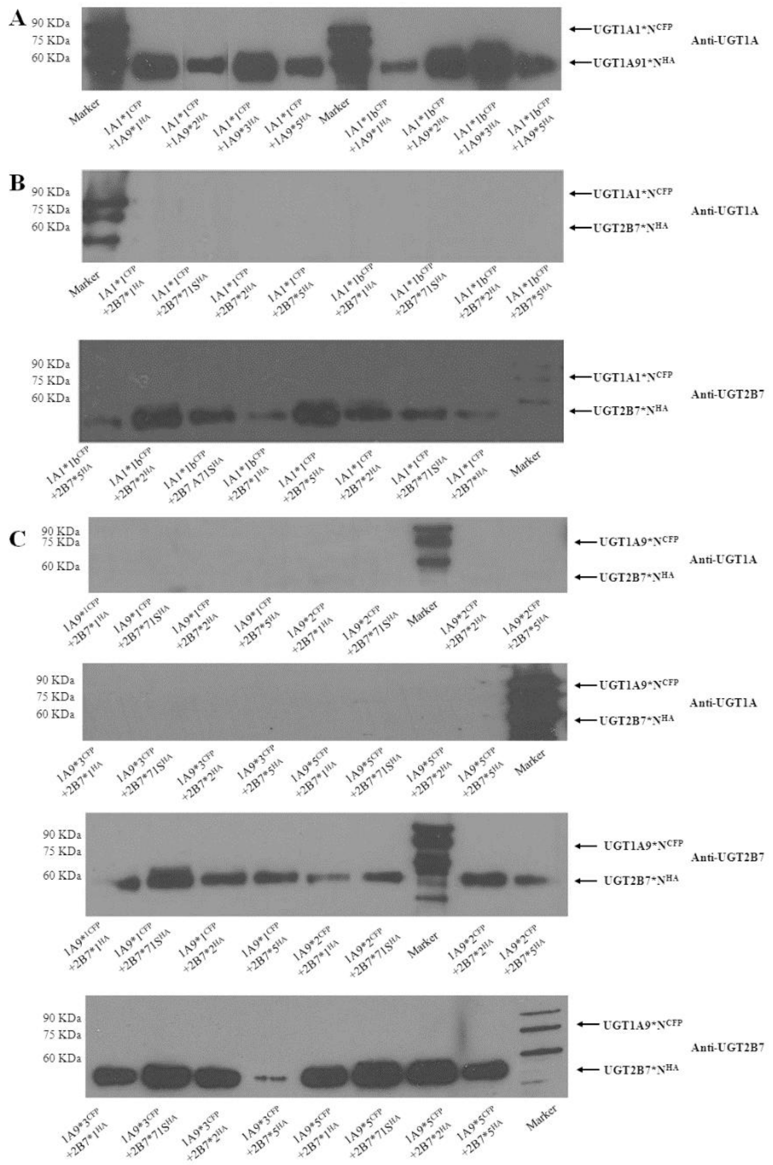

Figure S5. Co-IP analysis of mixtures of cell lysates from single expressed UGT1A1, UGT1A9 and UGT2B7 allozymes. Cell lysates were immunoprecipitated with anti-HA beads followed by Western blot analysis using anti-UGT1A and anti-UGT2B7 antibodies. Only HA-tagged proteins were detectable in the Western blot experiments, indicating no dimerization. (A) Mixtures of cell lysates from UGTA1*N^{CFP} and UGT1A9*N^{HA} single expression systems. (B) Mixtures of cell lysates from UGTA1*N^{CFP} and UGT2B7*N^{HA} single expression systems. (C) Mixtures of cell lysates from UGTA9*N^{CFP} and UGT2B7*N^{HA} single expression systems.

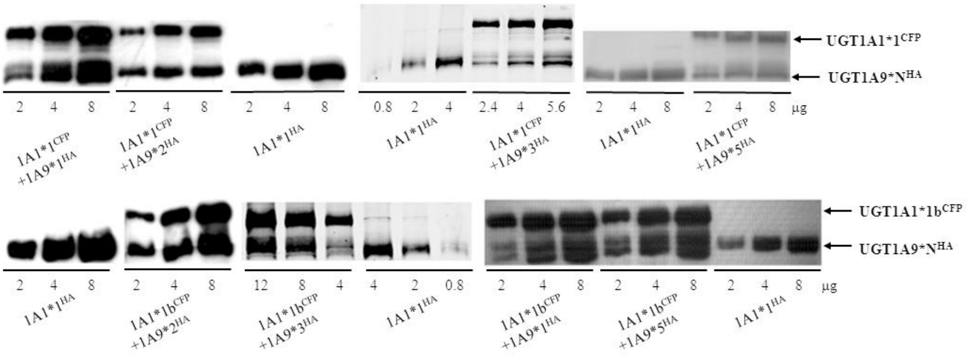

Figure S6. Immunoblot analysis of UGT1A1*N^{CFP}/1A9*N^{HA} co-expression systems. Total cell homogenates from Bac-to-Bac expression systems (0.8～16 μg) were subjected to 10% SDS-PAGE and further probed with anti-UGT1A antibodies.

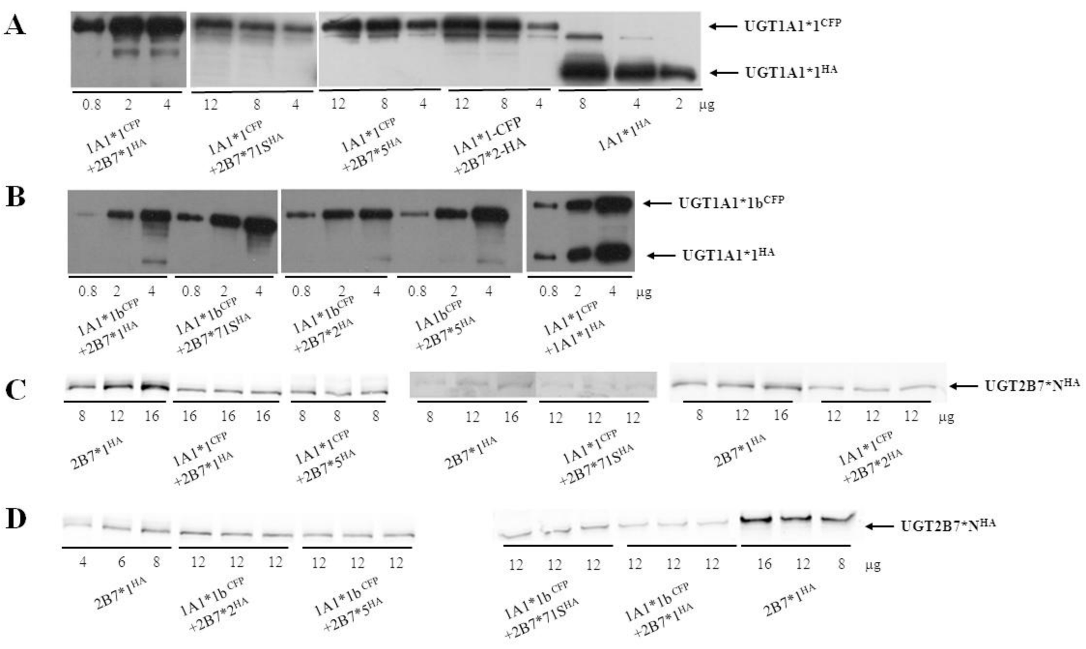

Figure S7. Immunoblot analysis of UGT1A1*N^{CFP} /2B7*N^{HA} co-expression systems. Total cell homogenates from Bac-to-Bac expression systems (0.8～16 μg) were subjected to 10% SDS-PAGE and the membranes were probed with anti-UGT1A antibodies (A and B) and anti-UGT2B7 antibodies (C and D), respectively.

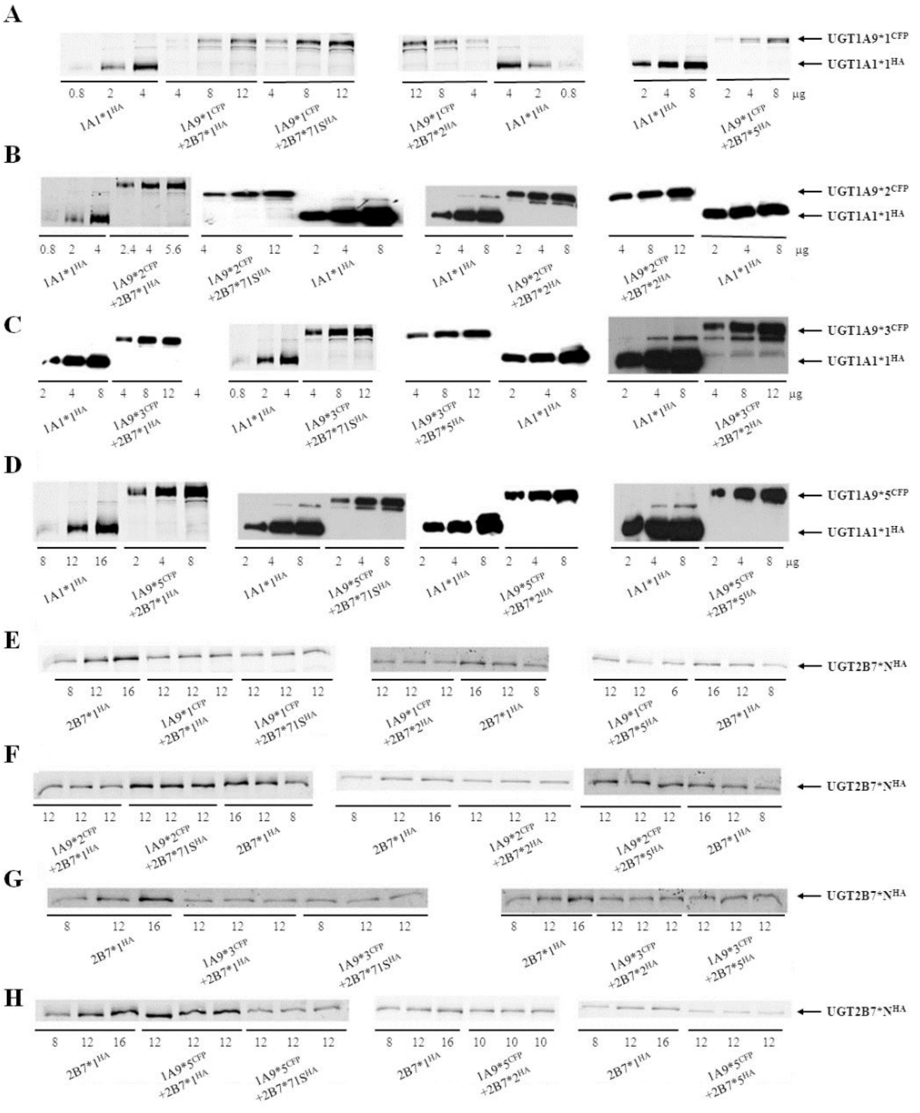

Figure S8. Immunoblot analysis of UGT1A9*N^{CFP}/2B7*N^{HA} co-expression systems. Total cell homogenates from Bac-to-Bac expression systems (0.8～16 μg) were subjected to 10% SDS-PAGE and the membranes were probed with anti-UGT1A antibodies (A, B, C and D) and anti-UGT2B7 antibodies (E, F G and H), respectively.

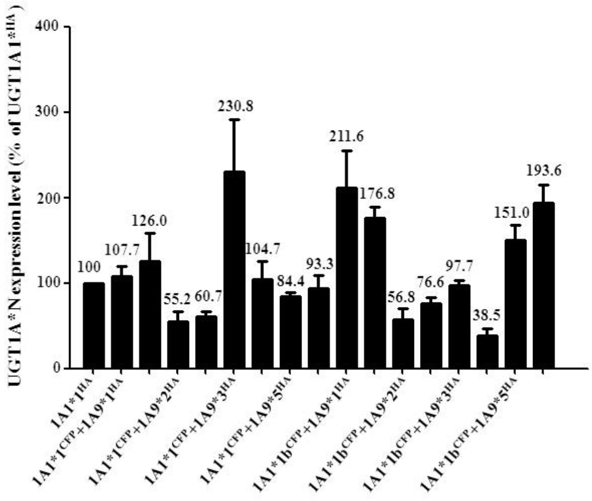

Figure S9. Relative expression levels of UGT1A1*N^{CFP} and UGT1A9*N^{HA} in UGT1A1*N^{CFP}/1A9*N^{HA} double expression systems. Quantity One 1-D analysis software was used for determination of relative expressions. Data are mean±SD from triplicate experiments.

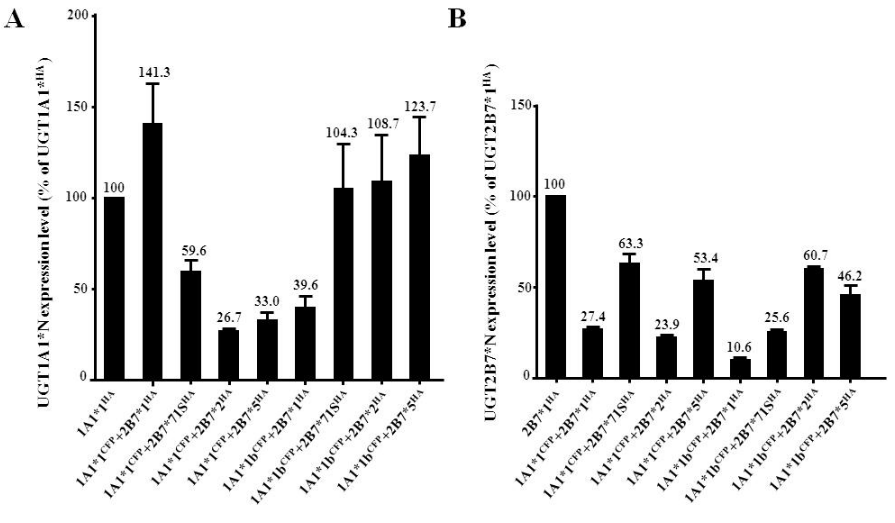

Figure S10. Relative expression levels of UGT1A1*N^{CFP} (A) and UGT2B7*N^{HA} (B) in UGT1A1*N^{CFP}/2B7*N^{HA} double expression systems. Quantity One 1-D analysis software was used for determination of relative expressions of UGT1A1*N^{CFP}. Quantity One 1-D analysis software and ODYSSEY Infrared Imaging software were used for determination of relative expressions of UGT2B7*N^{HA}. Data are mean±SD from triplicate experiments.

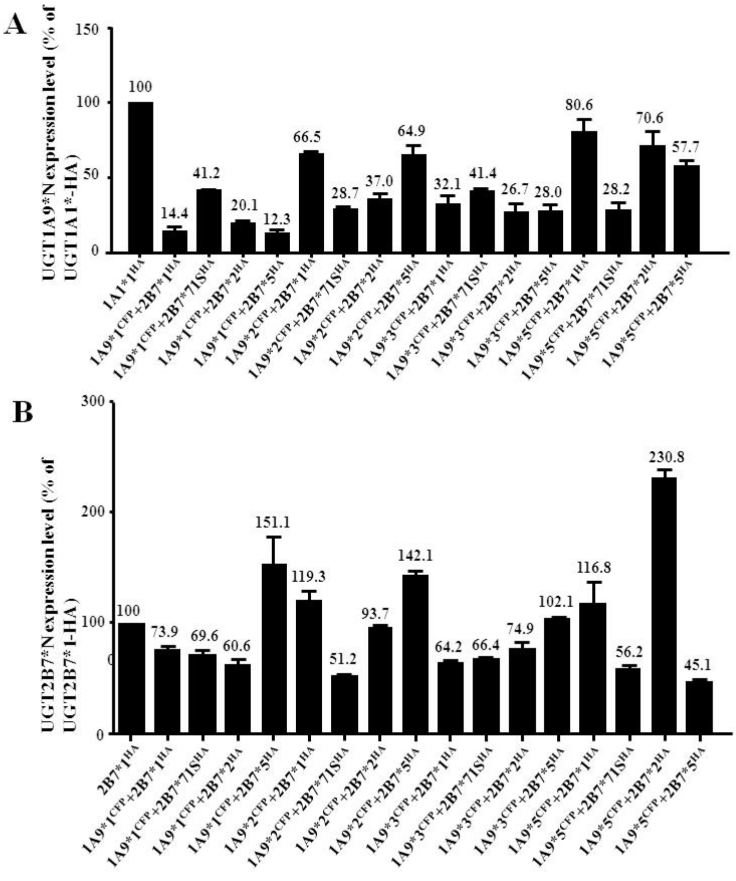

Figure S11. Relative expression levels of UGT1A9*N^{CFP} (A) and UGT2B7*N^{HA} (B) in UGT1A9*N^{CFP}/2B7*N^{HA} double expression systems. Quantity One 1-D analysis software was used for determination of relative expressions of UGT1A9*N^{CFP}. Quantity One 1-D analysis software and ODYSSEY Infrared Imaging software were used for determination of relative expressions of UGT2B7*N^{HA}. Data are mean±SD from triplicate experiments.

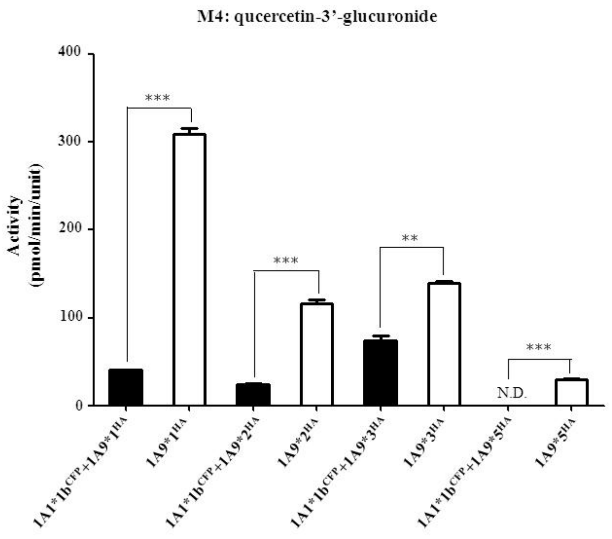

Figure S12. Enzyme activities for M4 (quercetin-3'-glucuronide) formation by UGT1A1*1b^{CFP}/1A9*N^{HA} co-expression systems. Data are mean±SD from triplicate experiments. Asterisks indicate statistically significant differences (***P<0.0001, **P<0.005, *P<0.05).

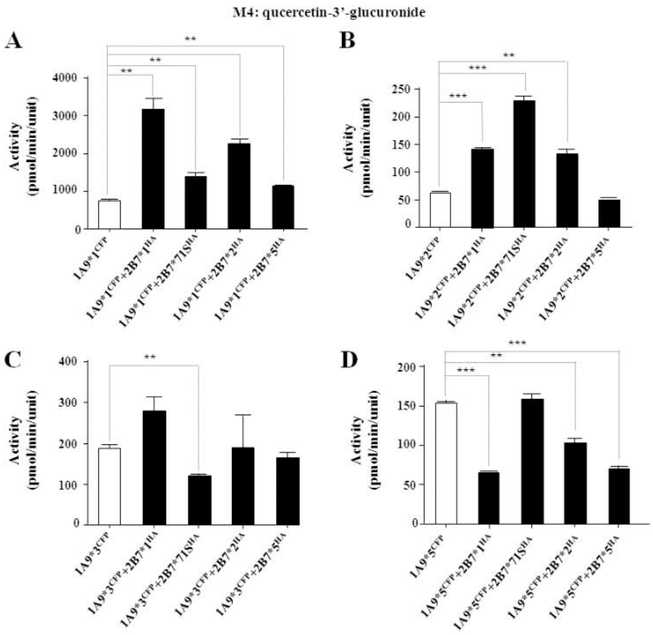

Figure S13. Enzyme activities for M4 (quercetin-3'-glucuronide) formation by UGT1A9*N^{CFP}/2B7*N^{HA} co-expression systems. Data are mean±SD from triplicate experiments. Asterisks indicate statistically significant differences (***P<0.0001, **P<0.005, *P<0.05).

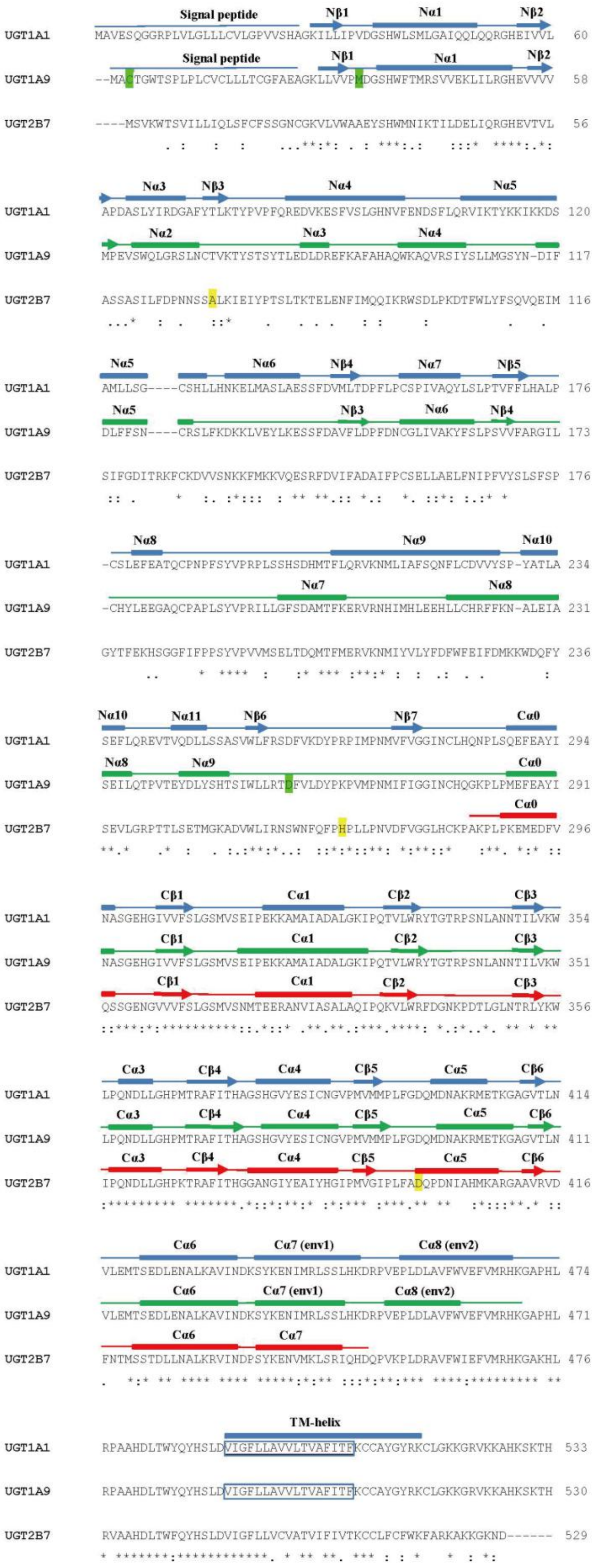

Figure S14. Multiple sequence alignment of UGT1A1, UGT1A9 and UGT2B7. The reported

UGT1A1 and UGT1A9 modeling secondary structures are in blue and green color. The secondary structures of UGT2B7 C-terminal domain by crystallography are in red. Mutation sites of UGT1A9 and UGT2B7 are highlighted with green and yellow, respectively. Putative transmembrane segments of UGT1A1 and UGT1A9 are framed. Identical amino acids are marked with * and homolog amino acid exchanges are marked with : or ..
